# Acceptability, feasibility and fidelity of an expanded role for community health workers for malaria elimination in Myanmar: A mixed-method study

**DOI:** 10.1371/journal.pgph.0004986

**Published:** 2025-08-13

**Authors:** Win Han Oo, Kaung Myat Khant, Ei Phyu Htwe, Win Htike, Nilar Aye Tun, May Chan Oo, Kaung Myat Thu, Aung Khine Zaw, Naw Hkawng Galau, Katherine O’Flaherty, Paul A. Agius, Freya J. I. Fowkes

**Affiliations:** 1 Health Security and Pandemic Preparedness Program, Burnet Institute, Melbourne, Victoria, Australia; 2 Centre for Epidemiology and Biostatistics, Melbourne School of Population and Global Health, University of Melbourne, Melbourne, Victoria, Australia; 3 Health Security and Malaria Program, Burnet Institute Myanmar, Yangon, Myanmar; 4 Disease Elimination Program, Burnet Institute, Melbourne, Victoria, Australia; 5 Department of Epidemiology and Preventive Medicine, Monash University, Melbourne, Victoria, Australia; 6 Faculty of Health, Deakin University, Melbourne, Victoria, Australia; University of Oxford, UNITED KINGDOM OF GREAT BRITAIN AND NORTHERN IRELAND

## Abstract

As countries transition to malaria elimination many are considering expanding the role of dedicated malaria community health workers (CHWs) to provide both malaria and limited primary health care services. The acceptability, feasibility and fidelity of an expanded role for CHW are vital factors for the successful implementation and sustainability of this health care model and data are needed to inform policy change.To further inform an expanded CHW model for malaria elimination, a mixed-method study nested in a trial which demonstrated effectiveness was undertaken to determine the acceptability, feasibility and fidelity of an expanded role for CHW in Myanmar. Data were collected as part of the trial including qualitative semi-structured in-depth interview with community leaders (n = 6) and health stakeholders (n = 14), focus group discussions (n = 36), supervision (n = 69) and field observation visits (n = 6) with CHWs. A quantitative cross-sectional survey was administered to community members (n = 643). Qualitative and quantitative data were analysed thematically and descriptively, and then triangulated for rigour. The expanded CHW model was acceptable to different levels of health stakeholders, CHWs and community members (97.4%, 626/643) because it addressed the community demand of health services (93.0%, 598/643) and promoted the roles of the CHWs within the framework of health regulations. Its implementation was also feasible due to the enthusiasm and high fidelity (97.1%, 67/69) of the CHWs as well as reliance of community members on expanded CHW services (98.3%, 632/643). However, aspects of training, supervision, supply chain management, referral mechanisms, and recording and reporting of data could have been improved. The expanded role for CHW model was found to be feasible to implement and acceptable to community members and stakeholders. With further investment and optimisation, wider-scale implementation of the model in malaria elimination settings may contribute to the goals of malaria elimination and effective primary health care in malaria elimination settings.

## Introduction

To decrease malaria burden and achieve malaria elimination, the core strategy of universally accessible malaria prevention, diagnosis and treatment should be strengthened to fill the gaps in malaria services [1]. In resource-limited countries, community health workers (CHWs) play a crucial role in strengthening this core strategy through prevention and case management of malaria in remote areas [2,3]. Various malaria CHW models have been designed and used primarily in malaria control and elimination programs in the Greater Mekong Subregion (GMS) and globally [4].

The GMS, comprising Cambodia, Lao PDR, Myanmar, Thailand, Vietnam and Yunnan Province of China, aims to eliminate malaria by 2030 [5], that is a reduction to zero incidence of indigenous malaria cases [6]. In the GMS, 146,718 confirmed malaria cases were reported in 2022, with 88% being from Myanmar [7]. More than 35,000 CHWs are being deployed across the GMS [8] and they provide malaria services such as administering rapid diagnosis test (RDT), treatment, referral to healthcare facilities and prevention including long-lasting insecticidal net distribution and behavioural change communication in underserved areas [4]. However, the proportion of malaria cases among febrile patients has declined with decreasing malaria transmission as the GMS progressed towards malaria elimination goal. Thus, the malaria CHWs and communities were less motivated to test for malaria due to low malaria positivity rates [9].

Consequently, GMS countries are developing or refining CHW models by expanding the role of malaria CHW into non-malaria health services in order to maintain CHW’s motivation for malaria blood examination and social role in the community [10]. Nevertheless, integration of CHWs into the national health system for broader primary health care services is still hampered by health system challenges including workforce related problems such as recognition of CHW as a health workforce underpinned by their quality of health care and associated training, supervision and monitoring challenges [11]. The expanded CHW model should provide malaria and additional primary health care services as per the demand of the community [12] but within which the national health system allows [13]. This means that the community will continue seeking malaria testing, an essential factor to maintain annual malaria blood examination rate required for malaria elimination certification, from the CHW as they will receive primary health care services even if they are not diagnosed as malaria.

Expanded CHW models developed and field implemented in malaria control setting in Myanmar proved to be effective in sustaining malaria blood examination rate [9] and reducing malaria burden of the country [14]; however, they were not endorsed by Ministry of Health [13]. Instead, the Ministry of Health deployed the Integrated Community Malaria Volunteer (ICMV) model as per political and logistical feasibility which provides primary health care services for malaria, dengue fever, tuberculosis (TB), lymphatic filariasis, Human Immunodeficiency Virus/ Acquired Immunodeficiency syndrome (HIV/AIDS) and leprosy since 2017–18 [15]. The existing expanded CHW models in Myanmar were developed as per logistic and administrative feasibility, and not co-designed with the community [12] and stakeholders including managers and policy makers [9,13].

A fit-for-purpose expanded CHW model should be evidence-based, effective in maintaining malaria blood examination rate, fulfilling the primary health care needs of the community and endorsed by policy makers and stakeholders. A recent trial in Myanmar evaluated the Community-delivered Integrated Malaria Elimination (CIME) model, an expanded CHW model co-designed with community members, community leaders, CHWs, and health stakeholders in Myanmar [12,13] and Lao PDR [16] in 72 CHW (S1 Table) serving villages in Yangon Region. This expanded CHW model provided services for malaria elimination and prevention, and pre-referral case management and assisted referral of malaria RDT-negative febrile illness, childhood diarrhoea, dengue fever and TB (S2 Table) [17]. While costing $206 per additional RDT conducted, compared to the current ICMV model, the CIME model increased village weekly malaria blood examination rate by a quarter and the village weekly referral rate for febrile illness, childhood diarrhoea, dengue fever and TB by more than three-fold [10].

While the trial demonstrated the effectiveness of the CIME model, assessment of its acceptability, feasibility and fidelity is still necessary for community utilisation and policy endorsement from stakeholders. The CIME model was assessed for its acceptability among the community members, CHWs, and health stakeholders, and feasibility to implement in the context of Myanmar, as well as fidelity defined as adherence of CHWs to performing their role in the model [18] measured by percentages of CIME CHWs adhering to CIME guidelines. Findings will inform the national scale-up of the CIME model in Myanmar and will have broad relevance of the region as other countries in the GMS consider expanding their CHW models [7].

## Materials and methods

### Ethics statement

The study was approved by Institutional Review Board, Myanmar Department of Medical Research, approval number (Ethics/DMR/2020/111) and Alfred Human Ethics Review Committee, Victoria, Australia (241/20). Written informed consents were obtained from all the study participants and their identity were kept confidential. To obtain informed consent from a participant, the data collector first explained all relevant study information and answered all questions raised by the participant. Afterwards, the informed consent was sought from the participant.

### Inclusivity in global research

Additional information regarding the ethical, cultural, and scientific considerations specific to inclusivity in global research is included in the Supporting Information (S1 Checklist)

### Study design and setting

The mixed-method study consisted of a community-based quantitative cross-sectional survey and qualitative data collection of focus group discussions (FGD), semi-structured in-depth interviews (IDI), and supervision and field observation conducted across the 69 CIME trial implementing villages in Hlegu, Kungyangon, and Taikkyi townships in Yangon Region of Myanmar from 1 January 2022 to 30 June 2022.

### Participant selection

In the trial, existing ICMVs in the included villages were enrolled in the study where they transitioned into CIME CHWs after getting the CIME training. During the study period, CHWs continued receiving the same amount in incentives (50,000 kyats [approximately 20 US$] per three months). For the survey, community members from these CIME implementing villages were engaged by the respective CHWs and selected approximately 8–10 community members per village from the sampling frame proportionately for age group and gender. To select CHWs to participate in qualitative data collection, the 69 CHWs who were enrolled in the trial were stratified into low and high performing groups according to their median monthly malaria blood examination rates during the trial. Then, in each township (n = 3), FGDs were performed with six high and six low performers (for a total 36 CHWs of 8 males and 28 females, median age of 36 years with interquartile range (IQR) from 31 to 45). Community leaders (n = 6) and local health stakeholders (n = 14) (malaria assistants, malaria investigators, lab assistants and midwives at community, township and regional levels) were purposively selected for IDIs based on their interest in community health, roles in the malaria program, experience with the community-delivered models, and their availability (S3 Table). Supervision for technical support as part of the project implementation was conducted for all 69 CHWs. For field observation, six CHWs (two CHWs per township for three townships) were selected purposively based on gender parity, monthly blood examination rates and accessibility to their residences at the time of data collection.

### Data collection

Following the political crisis (1 February 2021), there were some travel restrictions, so trained data collectors surveyed villagers via direct phone call in June 2022 using the pilot tested survey questionnaire (S1 Tool).

FGDs and IDIs were conducted in person in respective townships between March and June 2022 using pilot tested FGD facilitation guides and IDI topic guides (S2 Tool). Both FGDs and IDIs were audio recorded, and field notes were taken. At the end of each data collection day, the interviewers, facilitators, and note takers held a reflection session to assess data saturation determined by no additional data are being found whereby the researchers could develop themes and sub-themes of the category [19] and quality.

Structured non-participatory field observations of CHWs at their residences or clinics were made using a field observation checklist (S3 Tool) in April 2022. Staff from Yangon Regional Vector Borne Diseases Control Unit, Myanmar Ministry of Health conducted supervision visits for 69 CIME CHWs using a supervision checklist (S4 Tool) between January and April 2022.

### Data management and analysis

Categorical variables were summarised using frequency and percentage and numerical variables using mean and standard deviation or median and interquartile range where appropriate (S1-3 Files). Quantitative data were analysed using Stata 17. Qualitative audio data were transcribed verbatim, translated to English, and coded thematically (deductive, then inductive) by one in-country researcher, who is a researcher in the field of malaria and qualitative research, using NVivo 12. Themes, sub-themes, and thematic framework were produced with repetitive descriptive and analytical coding. Findings were presented thematically with key findings supported by direct data quotations. Qualitative research findings were triangulated with quantitative data. The findings were presented as per the domains of acceptability, feasibility and fidelity for components of the CIME model. This study is reported according to the Strengthening the Reporting of Observational Studies in Epidemiology (S2 Checklist) and Standards for Reporting Qualitative Research checklists (S3 Checklist).

## Results

Of the 643 surveyed community members, 54.5% were female (351/643) with a median age of 38 years (interquartile range 29–52). Only 3.3% of participants were mobile and migrant people (21/643) and the most common occupation was agriculture and livestock (43.2%, 278/643). Around a third of participants (32.5%, 209/643) had an under-five child in the family and had completed primary (33.1%, 213/643), middle (31.3%, 201/643) and high (20.5%, 132/643) school education (S4 Table). The 72 CHWs (S1 Table) recruited for the trial [10] had mean (SD) experience of 17.6 (0.2)years, working as CHW. They also had three years of malaria case-based surveillance experience.

### Role of the CHWs in the expanded model

#### Acceptability.

Overall, the expanded role for CHWs, the CIME model, was acceptable to communities, CHWs and health stakeholders. Health stakeholders in IDIs stated that they were satisfied with the CIME model because the CHWs actively participated in integrated primary healthcare activities and were reliable assistants in conducting these activities. Community leaders in IDIs reported that integrated services in the CIME model met the community needs because the CIME CHWs provided prompt care for common illnesses in the community free of charge.

According to the CHWs in FGDs, the CIME model’s integrated services for childhood diarrhoea and malaria RDT-negative febrile illness were of high demand in the community. By providing free-of-charge pre-referral treatment for those illnesses, the CHWs gained more community trust and social role, and felt that they were capable of community healthcare. Therefore, they preferred working as CIME CHWs over ICMV.

*“I like the CIME model more (than the ICMV). As a CIME CHW, I can provide more services to the community.” (CHW, Hlegu Township).*

Most survey participants (93.0%, 598/643) agreed that the CIME services met the community needs and having a CIME CHW in their village was convenient for their health (98.3%, 632/643). They expressed perceived satisfaction with the services provided by the CIME CHWs (97.7%, 628/643) particularly for the integrated childhood diarrhoea and RDT-negative febrile illness services (91.3%, 587/643). Therefore, almost every survey participant (97.4%, 626/643) showed acceptance and willingness to get the services from the CIME CHWs (Table 1).

**Table 1:** Acceptability of the CIME^*^ services by the community members by township (Survey).

Acceptability	Hlegu (N = 254)	Kungyangon (N = 170)	Taikkyi (N = 219)	Total (N = 643)
	n (%)	n (%)	n (%)	n (%)
**Satisfied with the services provided by the CIME CHW**				
**Agree**	249 (98.0)	166 (97.7)	213 (97.3)	628 (97.7)
**Don’t know**	3 (1.2)	3 (1.8)	3 (1.4)	9 (1.4)
**Disagree**	2 (0.8)	1 (0.6)	3 (1.4)	6 (0.9)
**The CIME CHW is skilful in providing health services**				
**Agree**	220 (86.6)	148 (87.1)	189 (86.3)	557 (86.6)
**Don’t know**	30 (11.8)	17 (10.0)	23 (10.5)	70 (10.9)
**Disagree**	4 (1.6)	5 (2.9)	7 (3.2)	16 (2.5)
**The services currently provided by the CIME CHW meet the needs of the community**				
**Agree**	235 (92.5)	163 (95.9)	200 (91.3)	598 (93.0)
**Don’t know**	18 (7.1)	6 (3.5)	13 (5.9)	37 (5.8)
**Disagree**	1 (0.4)	1 (0.6)	6 (2.7)	8 (1.2)
**It is convenient for us to have a CIME CHW in our village**				
**Agree**	251 (98.8)	169 (99.4)	212 (96.8)	632 (98.3)
**Don’t know**	3 (1.2)	1 (0.6)	5 (2.3)	9 (1.4)
**Disagree**	0 (0.0)	0 (0.0)	2 (0.9)	2 (0.3)
**Satisfied with the way the CIME CHW is communicating with the community**				
**Agree**	250 (98.4)	169 (99.4)	212 (96.8)	631 (98.1)
**Don’t know**	4 (1.6)	0 (0.0)	5 (2.3)	9 (1.4)
**Disagree**	0 (0.0)	1 (0.6)	2 (0.9)	3 (0.5)
**The CIME CHW is providing services with compassion**				
**Agree**	251 (98.8)	168 (98.8)	213 (97.3)	632 (98.3)
**Don’t know**	3 (1.2)	1 (0.6)	4 (1.8)	8 (1.2)
**Disagree**	0 (0.0)	1 (0.6)	2 (0.9)	3 (0.5)
**Satisfied with CIME CHW’s services for childhood diarrhoea and febrile illness**				
**Agree**	230 (90.6)	165 (97.1)	192 (87.7)	587 (91.3)
**Don’t know**	24 (9.5)	4 (2.4)	25 (11.4)	53 (8.2)
**Disagree**	0 (0.0)	1 (0.6)	2 (0.9)	3 (0.5)
**Accept and willing to get the same type of services from the CIME CHW in the future**				
**Agree**	249 (98)	168 (98.8)	209 (95.4)	626 (97.4)
**Don’t know**	4 (1.6)	2 (1.2)	8 (3.7)	14 (2.2)
**Disagree**	1 (0.4)	0 (0.0)	2 (0.9)	3 (0.5)

*Community-delivered Integrated Malaria Elimination model

According to FGDs, the febrile patients expected medication to relieve their symptoms when the malaria RDT result was negative. The CIME CHWs fulfilled their expectations by prescribing paracetamol. Similarly, CIME CHWs also provided oral rehydration solutions and zinc tablets for childhood diarrhoea. Additionally, antipyretics such as paracetamol were expensive and difficult to buy due to high demand during the COVID-19 pandemic. Therefore, the community members were satisfied with the free-of-charge fever treatment services of the CIME model. Community leaders and health stakeholders in IDIs also believed that free-of-charge prescription of medicines for pre-referral treatment of the integrated diseases in the CIME model made the community members accessible to relevant health services without out-of-pocket expense.

*“As I could prescribe medicine for other diseases, they (community members) trusted me that I would cure them even if the illness is not malaria.” (CHW, Hlegu Township)*

One community leader from IDIs mentioned that the CIME CHWs could deliver the patients to the right referral sites for both major and minor illnesses and voluntarily accompany the patients to the referral health centres. The CIME CHWs in FGDs also mentioned that the CIME referral form was helpful in referring the patients and tracing whether the patient went to the referral site. According to the community survey, the top reason for referral was RDT-negative fever (23.9%, 34/142) and almost all referred patients (97.2%, 138/142) were satisfied with the referral services provided by the CIME CHWs (S5 Table). The CIME CHWs in FGDs and health stakeholders in IDIs also accepted provision of assisted referral fees of 3,000 MMKs (approximately 1.4 USD at the time of implementation) because it could partially cover the transportation cost for poor patients and make them willing to go to the referred health facility.

*“(In the CIME model), I said that it was suspected dengue, and I would give transportation fees if you went to health centre. So, they liked the offer and went to health centre willingly.” (CHW, Taikkyi Township)*

Nevertheless, the CIME CHWs in FGDs and health stakeholders in IDIs suggested that the assisted referral fees in the CIME model were insufficient to fully cover transportation expenses for accessing health facilities located far from the patient residence considering the rising fuel costs for motorbike. Additionally, meal cost during the trip and healthcare expense at hospital or private clinic exacerbated this issue. The survey findings revealed that the CIME assisted referral fees (3,000 MMKs, approximately 1.4 USD), was lower than those of the median of other organisations (14,000 MMKs, approximately 6.7 USD) (S5 Table).

They also claimed that providing health education on common illnesses such as fever and diarrhoea improved community interest, trust and health seeking behaviour on the CIME services compared to the ICMV model.

*“When working as ICMV, we (the CHWs) talked about malaria, dengue and TB in health education sessions. Only three topics! But in the CIME, other febrile illnesses and diarrhoea are involved (in the health education sessions) … Compared to previous time (ICMV), more children visited me for the symptoms of sneezing or diarrhoea. As a CIME CHW, I feel delighted that they (community members) rely on me because of my health messages.” (CHW, Hlegu Township)*

Among the integrated disease services, malaria (46.8%, 301/643) was ranked as favourite service by highest proportion of participants (Table 2). In FGDs, CIME CHWs reported that community demand for malaria blood examination with RDT increased after working as CIME CHWs. Thus, the CIME model’s malaria blood examination service was acceptable to them. The CHWs in FGDs said that they could present their performance to their supervisors by recording detailed activities in the new CIME record book. The CHWs therefore gained more interest, confidence, and motivation in their activities.

**Table 2:** Awareness, preference and actual utilisation of different CIME services among community members (Survey).

Service provided diseases	Hlegu (N = 254)	Kungyangon (N = 170)	Taikkyi (N = 219)	Total (N = 643)
	n (%)	n (%)	n (%)	n (%)
**Community noticed health services provided by the CIME CHWs** ^**#**^				
**Malaria**	224 (88.2)	155 (91.2)	198 (90.4)	577 (89.7)
**Dengue**	139 (54.7)	131 (77.1)	140 (63.9)	410 (63.8)
**Childhood diarrhoea**	140 (55.1)	119 (70.0)	139 (63.5)	398 (61.9)
**RDT-negative fever**	114 (44.9)	75 (44.1)	111 (50.7)	300 (46.7)
**Tuberculosis**	120 (47.2)	58 (34.1)	104 (47.5)	282 (43.9)
Other diseases^†^	65 (25.6)	33 (19.4)	43 (19.6)	141 (21.9)
**Most popular health services in the community** ^**#**^				
**Malaria**	132 (52.0)	70 (41.2)	99 (45.2)	301 (46.8)
**Dengue**	34 (13.4)	47 (27.7)	52 (23.7)	133 (20.7)
**Childhood diarrhoea**	20 (7.9)	28 (16.5)	23 (10.5)	71 (11.0)
**RDT-negative fever**	18 (7.1)	13 (7.7)	25 (11.4)	56 (8.7)
**Tuberculosis**	29 (11.4)	4 (2.4)	18 (8.2)	51 (7.9)
**Other services**	14 (5.5)	4 (2.4)	4 (1.8)	22 (3.4)
**Receiving services from the CIME CHW**				
**Yes**	187 (73.6)	131 (77.1)	139 (63.5)	457 (71.1)
**No**	67 (26.4)	39 (22.9)	80 (36.5)	186 (28.9)

# Multiple response; ^†^Other disease includes maternal and child health services, non-communicable diseases, COVID-19, filariasis, HIV, leprosy.

The CHWs could also get supportive feedback from their supervisors in person through correction of errors in the reports at the monthly reporting meetings. Networking with peer CHWs in fortnightly gatherings enabled them to share good practices to overcome the challenges in implementing the CIME model. The health stakeholders in IDIs were also satisfied with the timeliness of reports submitted by the CIME CHWs.

#### Feasibility.

Regarding the feasibility of implementing and scaling up the CIME model, the CHWs in FGDs claimed that they could provide CIME services with minimal barriers because of community acceptance, CIME training, and medicines and commodities supplied during the CIME model implementation. Health stakeholders in IDIs believed that community trust and acceptance made the CIME model possible to implement in the future. Apart from malaria, health stakeholders in IDIs and the CIME CHWs in FGD claimed that there was no difficulty in performing the assessment for fever, diarrhoea, dengue and TB because of the training and medical supplies such as thermometers, paracetamol tablets or syrup and oral rehydration solution provided by the CIME model.

Nevertheless, restrictions on travelling, mass health education and community activities due to the COVID-19 pandemic and security concerns triggered by political crises hindered the implementation of the CIME model. Some CHWs from FGDs argued that they could not gather people for health talk because of COVID-19 pandemic and political instability. Additionally, they reported that some villagers were too busy to join the health education sessions. The CIME CHWs overcame these challenges by giving health education in a flexible manner such as educating the girls who gather near the lake to draw water or at the patient home with a small group of people visiting the patients.

Regarding feasibility of assigning malaria case investigation to CIME CHW, some health stakeholders in IDIs remarked that assigning the CHWs alone to execute case investigation might not be possible as it is a complex procedure. However, other stakeholders suggested that assigning case investigation to the CHWs could expand their interest and motivation in malaria elimination activities. Instead, all stakeholders recommended to assign CIME CHWs with preliminary malaria case investigation and classification which will help the malaria program staff with malaria elimination activities such as focus response activities of reactive case detection and community screening of malaria, treatment of cases, long-lasting insecticidal net distribution and indoor residual spraying.

Regarding the feasibility of the CIME referral service, the CIME CHWs in FGDs said that many patients who visited referral health centres experienced unavailability of centre staff because they were busy with other primary healthcare activities, such as vaccination. So, they had to go to a further health centre. Moreover, long waiting times at the hospital or rural health centre discouraged the patient from attending the referral sites. As a result, some patients preferred healthcare seeking from the nearest general practitioner or unqualified provider, or self-medication rather than visiting the health facility despite the referral services provided by the CIME CHWs. A health stakeholder in IDI also pointed out that many patients with fever or diarrhoea recovered from the illness with the pre-referral medication provided by the CIME CHWs and thus ended up not going to the referral site, thereby relying on the CIME CHW’s services.

FGDs also revealed issues in the referral fees reimbursement process practiced in the CIME model. When the referral form is filled by the CHW with relevant information about the patient, the patient takes this referral form to the healthcare provider from health centre, hospital or private clinic. The healthcare provider must fill in the diagnosis and affix their signature and stamp on the form. On return to the village, the CIME CHW who referred the patient checks the signature and stamp of the healthcare provider and then reimburses the patient with referral fees. Regardless of the procedure, some patients hesitated to ask the healthcare provider in health facility to sign and stamp on the CIME referral form. Even when they asked, some healthcare providers were reluctant to provide the necessary information, their signature and stamp on the referral forms, while others were not familiar with the referral form. As a result, the patients could not return signed and stamped referral forms with complete information to the CHWs. Thus, the CHWs were unable to reimburse the fees, which led to a loss of community trust in the referral services. To address this issue, some CHWs accompanied patients for their initial visit which made subsequent visits more convenient. However, the CHWs’ travel costs were not reimbursed.

Although the recording and reporting system of the CIME model was acceptable to the CIME CHWs and health stakeholders, and feasible to apply, there were some challenges in the recording system. The stock balance summary plot in carbonless malaria register designed to monitor stock balance was not comprehensive enough to check against the dynamic stock balance. The writing space available in the CIME record book was not enough for the CHWs to record all their activities. Some CHWs in FGDs mentioned that filling out numerous detailed records and fortnightly reporting requirement were burdensome. Additionally, some CHWs faced transportation difficulty to physically send the report to the assigned health centre. There were also misunderstanding among basic health staff in some health centres, so they did not accept the report from CIME CHWs and asked them to send the reports directly to township health department. Additionally, health stakeholders in IDIs still found data errors in the recording and reporting forms although the CIME CHWs checked data.

#### Fidelity.

Overall, the fidelity of the CIME model was demonstrated by CHW following the model guidelines and procedures. During supervision visits, it was found that almost every CHW (97.1%, 67/69) displayed the CIME signboard in a highly visible place to the public in accordance with the guidelines. CIME CHWs saw patients at a place with adequate ventilation (97.1%, 67/69), light (71.0%, 49/69) and privacy (82.6%, 57/69) (S6 Table). Supervision visits revealed that the CIME CHWs assessed the signs and symptoms of malaria (86.9%, 60/69) including the severe signs (82.6%, 57/69) and performed RDT testing (87.0%, 60/69). During RDT testing, > 95% of CIME CHWs were correct in preparing and administering RDTs and delivering RDT results. Supervision and field observation findings showed that the CIME CHWs filled data completely in the carbonless malaria register (98.6%, 68/69), ICMV daily register (88.4%, 61/69) and CIME record book (75.4%, 52/69), and accurately (91.3%, 63/69) in all the forms. They also submitted reports regularly (92.8%, 64/69) to assigned health centre (S7 Table).

The survey showed that majority of the participants (71.1%, 457/643) had received services from the CIME CHWs such as services for malaria RDT-negative febrile illness (56.9%, 260/457), malaria (28.2%, 129/457), childhood diarrhoea (15.3%, 70/457), dengue fever (9.4%, 43/457) and TB (2.6%, 12/457) during the CIME implementing period (Table 3). Most of the survey participants had received health education (79.4%, 511/643) during the implementing period. According to supervision findings, the CIME CHWs (n = 63) had conducted a median of 5 (2–11) health education sessions, 15 (10–27) participants per session, within two weeks before the supervision visit. The CIME CHWs also counselled individual patients, who visited them, about malaria RDT-negative febrile illness (97.1%, 67/69), childhood diarrhoea (86.9%, 60/69), dengue fever (82.6%, 57/69), malaria (79.7%, 55/69) and TB (79.7%, 55/69). The community survey showed that 22.1% of community members (142/643) received the referral service and 30% of them (42/142) received financial support to attend the referral health facility. Out of which, only 28.6% (12/42) of assisted referral fees was sourced from the CIME model (S5 Table).

**Table 3:** Utilisation of different CIME services among community members (Survey).

Services	Hlegu (N = 187)	Kungyangon (N = 131)	Taikkyi (N = 139)	Total (N = 457)
	n (%)	n (%)	n (%)	n (%)
**Services utilized** ^**#**^				
**Malaria**	45 (24.1)	44 (33.6)	40 (28.8)	129 (28.2)
**Dengue**	12 (6.4)	14 (10.7)	17 (12.2)	43 (9.4)
**Childhood diarrhoea**	30 (16.0)	26 (19.8)	14 (10.1)	70 (15.3)
**RDT-negative fever**	94 (50.3)	89 (67.9)	77 (55.4)	260 (56.9)
**Tuberculosis**	9 (4.8)	1 (0.8)	2 (1.4)	12 (2.6)
**Other diseases** ^**†**^	69 (36.9)	38 (29.0)	53 (38.1)	160 (35.0)
**Frequency of service utilisation over the implementation period of six months**				
**Median (IQR)**	2 (1-3)	2 (1-3)	2 (1-4)	2 (1-3)
**Range**	1-10	1-20	1-20	1-20

# multiple responses; ^†^Other diseases include non-communicable diseases, maternal and child health services and COVID-19.

### Management of CIME model implementation

#### Training.

The CHWs in FGDs claimed that the training improved their knowledge and skills for the CIME services, enabling them to analyse the clinical features of the illness not only for provisional diagnosis but also for disease severity.

*“Previously, we did not understand the signs and symptoms of the disease. Now, I understand well. I can differentiate what is serious and what is normal. Previously, I was confused and did not know what it was. Now, I understand clearly.” (CHW, Taikkyi Township)*

The CHWs in FGDs were also satisfied with the small group teaching method with four to five participants per training. They felt that trainers were patient and supportive, making the small group teaching interactive and effective for them. They also perceived that the presentations used in the training were visually attractive and simple for better understanding. Training Manual and Treatment Chart were written in Burmese and therefore convenient for them to follow.

FGDs revealed that contents of the training were also comprehensive, covering clinical features of the diseases in the model, practical procedures such as blood testing using RDT kits, measuring body weight and hand washing. They were also satisfied with getting refresher training for malaria, dengue and TB services, which had only been provided once since the beginning of the ICMV model.

The health stakeholders in IDIs believed that the CHWs became more proficient in performing healthcare activities after receiving the CIME training. They also suggested that the CIME CHWs could refer to the Training Manual for health services provision.

*“… Though they have already been proficient, they become more proficient now because of the training sessions. If there is something they forget, they will remember it by looking the Manual Book” (Health Stakeholders, Yangon Region)*

However, some CHWs from FGD had difficulties with the training schedule (four days was too short, with too few breaks), technical jargon in the training material as well as long commutes to training. To organise more effective CIME trainings, some CHWs and health staff suggested to include more interactive discussions, use less jargon, recruit younger persons as CIME CHWs and increase training days from four to five days.

#### Supply chain of medicine and commodities.

Supply chain system in the CIME model was acceptable to the health stakeholders and the CHWs. In supervision, all 69 CIME CHWs received initial supply of essential medicines and commodities. However, the CHWs and stakeholders highlighted ensuring uninterrupted supply of medicine is necessary for feasible implementation of the CIME model. The community leaders in IDIs said that the CIME CHWs did not receive enough medicine to provide the necessary services confirmed by supervision and field observation showing stock out of medicines and commodities in a few CHWs which included RDTs (7.3%, 5/69), paracetamol, zinc and ORS (11.6%, n = 8/69) (S8 Table). Supervision findings revealed that the CIME CHWs had fidelity to the stock management guidelines. As a result, RDT kit and medicine from majority of the CHWs (84.1%, n = 58/69) were well stored and ready to use (S9 Table).

## Discussion

The CIME model is a co-designed expanded CHW model for malaria elimination and primary healthcare. In Myanmar, its implementation was acceptable to CHWs, community members and different levels of health and community stakeholders because it addressed the community needs in primary healthcare and promoted the roles of the CHWs in the context of existing rules and regulations. CIME implementation was also feasible due to the enthusiasm of the CHWs and supply of necessary medicines and supplies. High fidelity of the CHWs to the CIME model implementation was also observed. However, there were barriers to the CIME model implementation such as COVID-19 pandemic associated restrictions, political instability and security concerns, limitation in training methodology and patient referral mechanism, and coordination with local health staff for recording and reporting that impacted essential health system components that affect CHW’s efficiency for improved healthcare services [20]. These barriers need to be addressed to successfully implement and scale up an expanded CHW model(s) to effectively contribute towards malaria elimination and primary health care in Myanmar and broadly in the GMS.

For the sustainability of a healthcare intervention model, community support and utilisation are vital. Community must be properly engaged during the design stage so that community, the end user, would have the sense of ownership over the community-delivered healthcare interventions [21]. The CIME model integrated community voices during the co-designing stage and included community-demanded health services [12]. As a result, the RDT-negative febrile illness and childhood diarrhoea services integrated in the CIME model were popular, appraised and used by the community members and therefore fitted in well with the community value system [22]. Consequently, the providers, CHWs, also felt satisfied as the services they provided were useful and liked by the community which strengthened provider-patient linkage and trust in the community; an essential supporting factor for sustainability and scale up of the CIME model. Similarly in other GMS countries, the CHWs were proud of providing health services, in addition to malaria, to their communities, satisfied with their work and willing to expand their role according to community needs [23,24].

The role of CHW in malaria prevention, diagnosis and treatment was universally endorsed by communities and stakeholders. But the additional assignment of investigating malaria cases for reactive surveillance and response strategies [25] to CHWs in the CIME model was controversial among the health stakeholders because there were concerns about accuracy of case investigation and classification made by a CHW. Malaria case investigation is a complex procedure even for a trained health staff and only a few of the basic health staff could classify all the six types of malaria cases correctly as per the Malaria Elimination Field Implementation Manual [26,27]. Nevertheless, a universal consensus among stakeholders was assigning CHWs for preliminary case investigation ahead of the official investigation and classification by the health staff. In this context, CHWs should be assigned for preliminary case investigation and classification supported by training and supervision. Accuracy, timeliness and completeness of the case investigation performed by CHW are yet to be assessed compared to those of health staff.

### The CIME model implementation

Although the CIME model in the current state is acceptable and feasible to implement, the implementation aspects of referral, recording and reporting, and coordination and communication at the field level, training and supply chain system that constitute health system elements of training and supervision, funding and governance require improvement [20].

#### Training and supervision.

The training for expanded CHW role within the CIME study was found to be not optimal in terms of training duration and methods and could be improved. The training should be useful and informative for the CHWs, and the skill acquired during the training should be applicable during service provision so that the CHWs will be motivated and serve well in the community [28]. Thus, the current training package and approach need to be reevaluated and redesigned to fit for the different backgrounds of current ICMVs if they are to be transformed into CIME CHWs. This should be complemented by optimal logistic support for CHWs who join the CIME training such as accommodation arrangement to ensure maximum learning of CHWs.

Notwithstanding that many CHWs felt satisfied with comprehensive data entry and showcasing their activities to the supervisors, some CHWs felt overburdened in data recording and reporting, fulfilling both CIME and ICMV models reporting requirements during the implementing period. If the CIME model is completely adopted in the future, the data burden could somehow decrease as CHWs will not need to record and report ICMV specific interventions. There were also misunderstandings between supervisors and CHWs regarding the reporting procedure. All these issues could be resolved when malaria and other CIME diseases’ case-based reporting is digitalized as the CHW will directly enter the data in the mobile phone application and the report will directly go the National Server accessible at different levels of the health system [29]. Nevertheless, another set of barriers such as inadequate internet access, software errors, and insufficient financial resources to support mobile phone-related costs may also need to be overcome [30].

#### Financing the model.

As reported in a recent systematic review, community members were found to be more encouraged to access health facilities if the financial support was sufficient to cover the cost of seeking healthcare [31]. The assisted referral fees provided in the CIME model was insufficient to cover the cost for health seeking at the referral health centre because of inflation associated increased healthcare and travel costs. In future implementation and scale up of the model, health financing component should be further scrutinised including the factors of patient affordability, willingness to pay, inflation rate and available budget for primary health care from domestic and international sources. Careful calculations and discussions to make inclusive informed decision regarding the amount and mechanism of assisted referral fees should be made given high amount of assisted referral fees could benefit the community members but burdensome for the available healthcare budget and ultimately impacts the cost-effectiveness of the model [10]. On the other hand, a minimum amount of referral fees should be provided in order to make all community members in need accessible to essential primary health care services, an essential pillar in the pathway to universal health coverage [32].

A study in Cambodia pointed out that the community acceptability could be undermined when the CHWs had poor social status among the community [23] hence CHWs should also be incentivized properly. The CHWs were also volunteers and they had to earn their living. Additionally, a study in Vietnam raised concerns regarding the expanded role, which could be a burden when there is excess workload from both the CHW services and their main jobs [24]. Inadequate incentives for their expanded role to reimburse their time could also reduce the motivation of the CHWs in their expanded role [23,24]. It is important to note that expanded CHW role could overburden the CHWs during the malaria outbreak season, as highlighted in a study in Thailand [33]. Heavy workload and inadequate compensations for the CHWs could decrease their satisfaction [33]. Appropriate recruitment, incentives and regular supportive supervision, essential health system factors for improved CHW’s efficiency [20], may increase the CIME CHWs’ motivation ensuring sustainability of expanded CHW models for malaria elimination.

#### Governance and organisational structure.

Some health staff from the referral health facility did not cooperate with the CIME referral system because they were not properly informed about the process of reimbursement for the CIME assisted referral fees. This led to issues in reimbursing assisted referral fees to the patients and hence damaged the community trust on the referral mechanism. In future CIME implementation, health staff should be well informed about the reimbursement process ahead of the field implementation. If the CIME model is adopted and integrated into the national health system, this problem may be solved as the patient visit to health facility following a referral by a CIME CHW could be checked in facility-based health records or on the National Health Online Database, when become available, directly by the health staff who supervise CIME model. As an interim solution before digitalisation of National Health Database, a CIME CHW may accompany the patient who is referred to the health centre and present the health staff so that cooperation and documentation at the health facility would improve. However, this approach could also cost travel expenses and time to the CIME CHW.

#### Medical products.

During the implementation of the expanded CIME CHW model, some CHWs experienced stock out of medicines and commodities. This could lead to decreased community trust and utilisation of services [34]. Since inclusion of services for RDT-negative febrile illness and diarrhoea improved overall community utilisation of the CIME model, careful management of supply chain for the integrated diseases to avoid stock-out is necessary for sustainability of community trust on the CIME model. Supply chain management should be based on retail actual usage of medicines to avoid stock out or overstocking. Supervisors should also place emphasis on stock report for an effective supply chain system.

### The CIME model in crisis

Overall, implementation of the CIME model encountered external barriers that could impact its sustainability and scale up nationwide. Political instability and armed conflict can impact the accessibility and delivery of community healthcare services [35], and the CIME model implementation is not an exception. While conflict impacts health services provision by any providers, using CHW network for primary health services provision is still a feasible option. With ongoing conflict, it becomes less feasible for the formal healthcare workforces to provide health services to the community because of resources limitation, loss of infrastructure, supply chain issues and security concerns. Amidst the conflict, the CIME CHWs could be a valuable resource to provide undisrupted health services to the community [36]. Unlike the traditional ICMV model, the CIME model is co-designed with community to meets the community health needs [12]. Additionally, with the suggestions of malaria program stakeholders, the CIME CHWs received training for common diseases in the community, regular supervision, supply of medicines and monetary or in-kind support [13]. Thus, the CIME CHWs can be effective in malaria control and primary health care in conflict-affected areas given the CHWs are from the local community and can move together with them [37]. Safety of the CHWs should be prioritised by providing safety training and limiting travel as much as possible [38]. Extensive training, regular stock replenishment and intensive supervision to support the CHWs are also necessary to optimise community healthcare during conflict [37–39]. In the context of Myanmar with expanding conflict in recent years, it is difficult to provide supervision and regular supply of essential medicines to the CHWs because of limited workforce at formal sector and vandalised infrastructure. In this situation, civil society organisations missioned for health and disease elimination may play a role in supporting the CHWs. Such organisations should engage with all stakeholders including the global and regional health actors, and local authorities for resilience of the primary health care system in conflict-affected areas. With strong partnerships among CHWs, civil society organisations and local authorities, reinforced by funding and technical support from international bodies, the health system in conflict areas could be strengthened to achieve malaria elimination and high coverage of primary health care.

Another important barrier encountered during the CIME implementation was COVID-19 pandemic that impacted CIME training, mass health education and other fieldworks. As the COVID-19 is no longer a public health emergency of international concern since 5 May 2023 [40], any future CIME model implementation and scale-up may not be impacted by COVID-19 threat although the virus is still widespread globally. Taking the lesson learned from CIME model implementation, future implementation and scale-up of the CIME model could be prepared for the threat of emerging and re-emerging infectious diseases. Given the inclusion of pre-referral fever management and assisted referral in the CIME model, the model could also be instrumental in responding emerging infectious diseases which may be the major concern of community and health stakeholders when emerged [41].

## Limitations

During the supervision and field observation visit, the supervisors and research team members could not observe case management of all diseases in the CIME model because of the time constraint, limited cases presented to CIME CHWs and security concerns. Therefore, some CHWs were asked about case management of some diseases such as TB in simulation and their answers were entered into the checklist which may not reflect the actual treatment practice of the CIME CHWs. Additionally, CHWs assisted in participant recruitment which could have led to bias with recruitment of community members who were more satisfied with CHW services and random sampling may have been more representative of the population. The survey was conducted via phone calls to participants due to political instability which could lead to selection bias excluding participants without phone access. This potential selection bias was mitigated by instructing CHWs to make appointment with the potential participants without phone access and giving their mobile phone to them to answer the call from the data collector. CHWs were also instructed not to stay with the participants during the call for data collection considering their privacy and confidentiality. CHWs were later reimbursed with mobile phone credit to compensate it. Data collection was only done in Yangon Region where the CIME model was implemented and hence the findings may not be generalizable to other states and regions in Myanmar, with different geographical, socio-demographic and ethnic backgrounds and health system capacity. Similar studies should be implemented in remote, ethnic, or conflict-affected regions in the future for broader generalizability if logistic and administrative requirements are met.

## Conclusions

The effective expanded CIME CHW model met the community needs and was deemed acceptable and feasible to implement in malaria elimination and primary healthcare settings in Myanmar. Optimised training, supportive supervision, uninterrupted supply of medicines, digitalisation of recording and reporting system, improved case referral mechanism and full recognition and integration of CIME CHWs into the health system to ensure the CHWs’ competency and adherence to the guidelines are the key areas to be strengthened for successful implementation of the CIME model in the future (Table 4). Along with further investments and optimisations, the CIME model should be scaled up as it is effective and feasible to implement due to the acceptance of community members and stakeholders, and adherence of the CIME CHWs. Other GMS countries may learn lessons from CIME model implementation in Myanmar and adopt it for their health system context so that the CIME model will contribute towards the regional malaria elimination goal and primary health care in the GMS.

**Table 4:** Summary of recommendations.

	Issues	Recommendations
**Training**	• Short training duration for large volume of training contents • Technical jargon in the training	• Re-evaluation and redesigning of current training package and approach • Optimal logistic support for the CHWs to join the training such as accommodation arrangement
**Supervision**	• Errors in data reporting to supervisors • Suboptimal layout of the reporting form • Transportation difficulty and associated cost for reporting • Misunderstanding between CHWs and supervisors regarding reporting procedure	• Practicing peer data checking and correction before report submission • Digitalisation of recording and reporting
**Financing**	• Inadequate assisted referral fees • Decreasing motivation of CHWs due to increased workload	• Further assessment, calculation and decision for assisted referral fees and mechanism • Appropriate recruitment, incentives and supportive supervision
**Governance**	• Unawareness and non-cooperation of health staff at the referral sites	• Adoption and integration of the CIME model into the national health system • Digitalisation of the National Health Database
**Medical products**	• Stockout of medicines and commodities	• Supply chain management based on actual retail of products • Supervision emphasized on stock report to ensure effective supply chain system
**Crisis**	• Political instability and security concerns	• Engagement with all stakeholders by civil society organisations • Strong partnership among the CHWs, civil society organisations and authorities • Funding and technical support from international organisations

CIME - Community-delivered Integrated Malaria Elimination model

CHW - Community Health Worker

## Supporting information

S1 Checklist Inclusivity in global research. (DOCX)

S2 Checklist STROBE checklist. (DOCX)

S3 Checklist SRQR checklist. (DOCX)

S1 File Analytic codes. (DO)

S2 File Supervision dataset. (DTA)

S3 File Survey dataset. (DTA)

S1 Table Background characteristics of the CHWs. (DOCX)

S2 Table Summary of ICMV and CIME models. (DOCX)

S3 Table Participants in qualitative discussions. (DOCX)

S4 Table Surveyed community members. (DOCX)

S5 Table Referral services (survey). (DOCX)

S6 Table Clinic setting (supervision). (DOCX)

S7 Table Records and registers (supervision). (DOCX)

S8 Table Stock out (supervision). (DOCX)

S9 Table Storage (supervision). (DOCX)

S1 Tool. Questionnaire for cross-sectional survey. (PDF)

S2 Tool. Interview and FGD guides. (PDF)

S3 Tool. Field observation checklist. (PDF)

S4 Tool. Supervision checklist. (PDF)
