# Supplementary material for: Acceptability, feasibility and fidelity of an expanded role for community health workers for malaria elimination in Myanmar: A mixed-method study
Source: PLOS Glob Public Health. 2025 Aug 13;5(8):e0004986. doi: 10.1371/journal.pgph.0004986 (PMC12349089; doi:10.1371/journal.pgph.0004986)
Supplement: S2 Checklist — (DOCX) [file pgph.0004986.s002.docx]

**S2 Checklist - STROBE checklist**

|  | Item No. | Recommendation | Page  No. | Relevant text from manuscript |
| --- | --- | --- | --- | --- |
| **Title and abstract** | 1 | (*a*) Indicate the study’s design with a commonly used term in the title or the abstract | 1 | a mixed-method study |
|  |  | (*b*) Provide in the abstract an informative and balanced summary of what was done and what was found | 2 | a mixed-method study to determine the acceptability, feasibility and fidelity of an expanded role for CHW, including qualitative semi-structured in-depth interview with community leaders (n=6) and health stakeholders (n=14), focus group discussions (n=36), supervision (n=69) and field observation visits (n=6) with CHWs. A quantitative cross-sectional survey Qualitative and quantitative data were analysed thematically and descriptively.  The expanded role for CHW model was found to be feasible to implement and acceptable to community members and stakeholders |
| Introduction | | | |  |
| Background/rationale | 2 | Explain the scientific background and rationale for the investigation being reported | 3-5 | The existing expanded CHW models in Myanmar were developed as per logistic and administrative feasibility, and not co-designed  Expanded CHW models developed and field implemented in malaria control setting in Myanmar proved to be effective in sustaining malaria blood examination rate  A fit-for-purpose expanded CHW model should be evidence-based, effective in maintaining malaria blood examination rate, fulfil the primary health care needs of the community and endorsed by policy makers and stakeholders.  This expanded CHW model provided services for malaria elimination and prevention, and pre-referral case management and assisted referral of malaria RDT-negative febrile illness, childhood diarrhoea, dengue fever and TB  While the trial demonstrated the effectiveness of the CIME model, assessment of its acceptability, feasibility and fidelity is still necessary for community utilisation and policy endorsement from stakeholders. |
| Objectives | 3 | State specific objectives, including any prespecified hypotheses | 5 | The CIME model was assessed for its acceptability among the community members, CHWs, and health stakeholders, and feasibility to implement in the context of Myanmar, as well as fidelity defined as adherence of CHWs to performing their role in the model |
| Methods | | | |  |
| Study design | 4 | Present key elements of study design early in the paper | 6 | community-based quantitative cross-sectional survey and qualitative data collection of focus group discussions (FGD), semi-structured in-depth interviews (IDI), and supervision and field observation |
| Setting | 5 | Describe the setting, locations, and relevant dates, including periods of recruitment, exposure, follow-up, and data collection | 6 | across the 69 CIME trial implementing villages in Hlegu, Kungyangon, and Taikkyi townships in Yangon Region of Myanmar from January to June 2022. |
| Participants | 6 | (*a*) *Cohort study*—Give the eligibility criteria, and the sources and methods of selection of participants. Describe methods of follow-up  *Case-control study*—Give the eligibility criteria, and the sources and methods of case ascertainment and control selection. Give the rationale for the choice of cases and controls  *Cross-sectional study*—Give the eligibility criteria, and the sources and methods of selection of participants | 6, 7 | community members from the CIME implementing villages  approximately 8-10 community members per village proportionately for age group and gender  Supervision for technical support as part of the project implementation was conducted for all 69 CHWs |
|  |  | (*b*) *Cohort study*—For matched studies, give matching criteria and number of exposed and unexposed  *Case-control study*—For matched studies, give matching criteria and the number of controls per case |  |  |
| Variables | 7 | Clearly define all outcomes, exposures, predictors, potential confounders, and effect modifiers. Give diagnostic criteria, if applicable | 8 | The primary outcome (acceptability and willingness to receive services from the expanded CHW model) |
| Data sources/ measurement | 8* | For each variable of interest, give sources of data and details of methods of assessment (measurement). Describe comparability of assessment methods if there is more than one group | 7 | trained data collectors surveyed villagers via direct phone call in June 2022 using the pilot tested survey questionnaire |
| Bias | 9 | Describe any efforts to address potential sources of bias | - | - |
| Study size | 10 | Explain how the study size was arrived at | 6, 7 | selected approximately 8-10 community members per village  643 surveyed community members  Supervision for technical support as part of the project implementation was conducted for all 69 CHWs |

Continued on next page

| Quantitative variables | 11 | Explain how quantitative variables were handled in the analyses. If applicable, describe which groupings were chosen and why | NA | NA |
| --- | --- | --- | --- | --- |
| Statistical methods | 12 | (*a*) Describe all statistical methods, including those used to control for confounding | 8 | categorical variables were summarised using frequency and percentage  The numerical variables were summarised using mean and standard deviation for normal distribution or median and interquartile range for skewed data |
|  |  | (*b*) Describe any methods used to examine subgroups and interactions | NA | NA |
|  |  | (*c*) Explain how missing data were addressed | - | Presence of missing values are mentioned in the tables |
|  |  | (*d*) *Cohort study*—If applicable, explain how loss to follow-up was addressed  *Case-control study*—If applicable, explain how matching of cases and controls was addressed  *Cross-sectional study*—If applicable, describe analytical methods taking account of sampling strategy | - | Formal sample size or power calculation is not done |
|  |  | (*e*) Describe any sensitivity analyses |  |  |
| Results | | | | |
| Participants | 13* | (a) Report numbers of individuals at each stage of study—eg numbers potentially eligible, examined for eligibility, confirmed eligible, included in the study, completing follow-up, and analysed | 6,7 | 643 surveyed community members  69 CHWs |
|  |  | (b) Give reasons for non-participation at each stage | NA | NA |
|  |  | (c) Consider use of a flow diagram | NA | NA |
| Descriptive data | 14* | (a) Give characteristics of study participants (eg demographic, clinical, social) and information on exposures and potential confounders | 6 | Of the 643 surveyed community members, 55% were female (351/643) and they had a median age of 38 years with an interquartile range (29 to 52). Only 3.3% of participants were mobile and migrant people (21/643) and the most common occupation was agriculture and livestock (43%, 278/643). Around a third of participants (33%, 209/643) had an under-five child in the family and had completed primary (33%, 213/643), middle (31%, 201/643) and high (21%, 132/643) school education |
|  |  | (b) Indicate number of participants with missing data for each variable of interest | NA | NA |
|  |  | (c) *Cohort study*—Summarise follow-up time (eg, average and total amount) | NA | NA |
| Outcome data | 15* | *Cohort study*—Report numbers of outcome events or summary measures over time |  |  |
|  |  | *Case-control study—*Report numbers in each exposure category, or summary measures of exposure |  |  |
|  |  | *Cross-sectional study—*Report numbers of outcome events or summary measures | 9-10 | almost every survey participant (97.4%, 626/643) showed acceptance and willingness to get the services from the CIME CHWs |
| Main results | 16 | (*a*) Give unadjusted estimates and, if applicable, confounder-adjusted estimates and their precision (eg, 95% confidence interval). Make clear which confounders were adjusted for and why they were included | NA | NA |
|  |  | (*b*) Report category boundaries when continuous variables were categorized | NA | NA |
|  |  | (*c*) If relevant, consider translating estimates of relative risk into absolute risk for a meaningful time period | NA | NA |

Continued on next page

| Other analyses | 17 | Report other analyses done—eg analyses of subgroups and interactions, and sensitivity analyses | NA | NA |
| --- | --- | --- | --- | --- |
| Discussion | | | | |
| Key results | 18 | Summarise key results with reference to study objectives | 20 | Its implementation was acceptable to CHWs, community members and different levels of health and community stakeholders because it addressed the community needs in primary healthcare and promoted the roles of the CHWs in the context of existing rules and regulations. CIME implementation was also feasible due to the enthusiasm of the CHWs and supply of necessary medicines and supplies. High fidelity of the CHWs to the CIME model implementation was also observed. |
| Limitations | 19 | Discuss limitations of the study, taking into account sources of potential bias or imprecision. Discuss both direction and magnitude of any potential bias | 26 | the supervisors and research team members could not observe case management of all diseases in the CIME model because of the time constraint, limited cases presented to CIME CHWs and security concerns.  Additionally, CHWs assisted in participant recruitment which could have led to bias with recruitment of community members who were more satisfied with CHW services. |
| Interpretation | 20 | Give a cautious overall interpretation of results considering objectives, limitations, multiplicity of analyses, results from similar studies, and other relevant evidence | 26 | It also met the community needs and was deemed acceptable and feasible to implement in malaria elimination and primary healthcare settings in Myanmar. |
| Generalisability | 21 | Discuss the generalisability (external validity) of the study results | 26 | Data collection was only done in Yangon Region where the CIME model was implemented and hence the findings may not be generalisable to other states and regions in Myanmar with different geographical, sociodemographic and ethnic backgrounds and health system capacity. |
| Other information | |  | | |
| Funding | 22 | Give the source of funding and the role of the funders for the present study and, if applicable, for the original study on which the present article is based | - | Submission form of the journal |

*Give information separately for cases and controls in case-control studies and, if applicable, for exposed and unexposed groups in cohort and cross-sectional studies.

**Note:** An Explanation and Elaboration article discusses each checklist item and gives methodological background and published examples of transparent reporting. The STROBE checklist is best used in conjunction with this article (freely available on the Web sites of PLoS Medicine at http://www.plosmedicine.org/, Annals of Internal Medicine at http://www.annals.org/, and Epidemiology at http://www.epidem.com/). Information on the STROBE Initiative is available at www.strobe-statement.org.
