# Supplementary material for: Acceptability, feasibility and fidelity of an expanded role for community health workers for malaria elimination in Myanmar: A mixed-method study
Source: PLOS Glob Public Health. 2025 Aug 13;5(8):e0004986. doi: 10.1371/journal.pgph.0004986 (PMC12349089; doi:10.1371/journal.pgph.0004986)
Supplement: S1 Table — (DOCX) [file pgph.0004986.s007.docx]

**S1 Table: Background characteristics of Community Health Workers (CHW)**

| **Characteristics** | **Hlegu**  **(N=31)** | **Kungyangon**  **(N=17)** | **Taikkyi**  **(N=24)** | **Total**  **(N=72)** |
| --- | --- | --- | --- | --- |
|  | n (%) | n (%) | n (%) | n (%) |
| **Completed age in years** | | | | |
| **Median (IQR)** | 41.0 (35.0-49.0) | 41.0 (32.0-50.0) | 39.0 (29.0-46.0) | 40.5 (31.0-50.0) |
| **Gender** | | | | |
| **Female** | 15 (48.4) | 15 (88.2) | 18 (75.0) | 48 (66.7) |
| **Male** | 16 (51.6) | 2 (11.8) | 6 (25.0) | 24 (33.3) |
| **Experience as CHW (years)** | | | | |
| **Mean (SD)** | 17.7 (0.2) | 17.7 (0.1) | 17.6 (0.1) | 17.6 (0.2) |
| **Experience with malaria case-based reporting (years)** | | | | |
| **Mean (SD)** | 3.0 (0.2) | 3.0 (0.1) | 3.0 (0.1) | 3.0 (0.2) |
