# Supplementary material for: Acceptability, feasibility and fidelity of an expanded role for community health workers for malaria elimination in Myanmar: A mixed-method study
Source: PLOS Glob Public Health. 2025 Aug 13;5(8):e0004986. doi: 10.1371/journal.pgph.0004986 (PMC12349089; doi:10.1371/journal.pgph.0004986)
Supplement: S2 Table — (DOCX) [file pgph.0004986.s008.docx]

**S2 Table: Summary of ICMV and CIME models**

| Disease | ICMV | CIME |
| --- | --- | --- |
| Malaria | ➢ Prevention and health education, community mobilisation for malaria activities ➢ Helping in distribution of long-lasting insecticidal nets and dipping existing bed nets ➢ Early diagnosis, treatment and referral of malaria cases according to the National Malaria Treatment Guidelines  ➢ Early warning and reporting of possible malaria outbreaks in the community to the health department  ➢ Data entry, compilation and reporting of rapid diagnostic test-tested malaria cases using the prescribed formats  ➢ Helping in entomological, malaria elimination and community-based research activities | Interventions in the ICMV Model to be continued in the CIME Model   - Malaria Diagnosis using RDT - Treatment and/or assisted referral of malaria cases according to the national malaria treatment guideline of Myanmar - Prevention interventions   - Behavioral change communication activities,   - Assistance in long lasting insecticidal nets and mosquito repellent distribution   - Community mobilization works - Contribution in stratification of malaria situation by malaria transmission intensity, and in surveillance and research activities |
|  |  | Interventions to be modified in transition into the CIME Model   - Reporting of malaria cases within 24 hours of diagnosis using direct phone call and/or text message to their immediate supervisor/ local vector borne diseases control programme  (VBDC) staff - Serving as the directly observed treatment providers |
|  |  | New interventions to be added in transition into the CIME Model   - Conducting preliminary case investigation procedures and provide provisional identification of malaria cases to differentiate indigenous and imported cases - Assisting case and foci investigation, and response teams in community mobilization, information collection and larval source management activities - Serving as the translators for the case and foci investigation teams in areas where people use different languages other than Burmese |
| Dengue | ➢ Assisting Vector Borne Disease Control Program staff and basic health staff (BHS) in vector control activities  ➢ Helping in referral of dengue suspected patients to the nearest health centre | Interventions in the ICMV Model to be continued in the CIME Model   - Assisting VBDC program staff and basic health staff (BHS) in dengue prevention and vector control activities - Referral of dengue suspected patients to the nearest health centre - Serving as a role model for dengue prevention and vector control activities in the community using positive deviance approach |
| Tuberculosis (TB) | ➢ Checking for TB signs and symptoms, and referral of suspected TB patients  ➢ Contact tracing of TB patients in their communities  ➢ Serving as directly observed treatment providers  ➢ Following up the lost-to-follow-up TB patients (defaulter tracing)  ➢ Helping TB patients in follow-up sputum examinations  ➢ Assisting BHS in TB health education talks and active case detection activities | Interventions in the ICMV Model to be continued in the CIME Model   - Detection and referral of TB suspected patients by checking TB sign and symptoms - Contact tracing of TB patients in their community - Serving as the directly observed treatment providers - Following up the lost-to-follow-up TB patients (defaulter tracing) - Helping TB patients in follow up sputum examinations - Assisting BHSs in TB health education talks and active case detection activities |
| Lymphatic filariasis | ➢ Helping BHS in mass drug administration activity for lymphatic filariasis elimination  ➢ Reporting of lymphatic filariasis cases to the health department and assisting in the home-based care of lymphatic filariasis cases | Not included in the CIME model |
| Human Immunodeficiency Virus / Acquired Immunodeficiency Syndrome (HIV/ AIDS) | ➢ Providing health education on HIV/AIDS and other sexually transmitted diseases  ➢ Assisting in the mitigation of discrimination against HIV/AIDS patients  ➢ Informing villagers of locations of clinics where they can get free services for HIV/AIDS and other sexually transmitted diseases  ➢ Helping in referral of clients who need sexually transmitted disease treatment and HIV testing (Note: ICMVs must keep HIV/AIDS and sexually transmitted diseases information confidential.) | Not included in the CIME model |
| Leprosy | ➢ Providing health education in the community – communicating key leprosy messages to villagers  ➢ Referral of suspected leprosy cases to health departments  ➢ Referral of disabled, old and new leprosy patients who are suffering from reaction and complications of leprosy  ➢ Assisting BHS and leprosy program staff to detect new leprosy cases  ➢ Assisting the leprosy program in its public health projects | Not included in the CIME model |
| Childhood diarrhoea | Not included in the ICMV model | New interventions to be added in transition into the CIME Model   - Prevention - Health education and water sanitation, and hygiene promotion - Diagnosis - Clinical diagnosis and dehydration assessment - Treatment and referral - Rehydration therapy using oral rehydration solution and oral zinc tablet - Assisted referral to health centres |
| Malaria RDT-negative febrile illness | Not included in the ICMV model | New interventions to be added in transition into the CIME Model   - Prevention and health education - Symptomatic treatment with antipyretics and immediate assisted referral |

**Glossary of abbreviations and terms in this table**

**BHS -** Basic Health Staff

**CIME -** Community-delivered Integrated Malaria Elimination

**HIV/AIDS -** Human immunodeficiency virus / acquired immunodeficiency syndrome

**ICMV -** Integrated Community Malaria Volunteer

**RDT** - Rapid diagnostic test

**TB** - Tuberculosis

**VBDC -**Vector Borne Diseases Control Programme
