# Supplementary material for: Acceptability, feasibility and fidelity of an expanded role for community health workers for malaria elimination in Myanmar: A mixed-method study
Source: PLOS Glob Public Health. 2025 Aug 13;5(8):e0004986. doi: 10.1371/journal.pgph.0004986 (PMC12349089; doi:10.1371/journal.pgph.0004986)
Supplement: S3 Table — (DOCX) [file pgph.0004986.s009.docx]

S3 Table: Background characteristics of participants in qualitative discussions

| **Characteristics** | **Focus group discussion with community health workers**  **(N=36)** | **Semi-structured interviews with community leaders**  **(N = 6)** | **Semi-structured interviews with health stakeholders (N=14)** |
| --- | --- | --- | --- |
|  | n (%) | n (%) | n (%) |
| **Age (years)** |  |  |  |
| **<= 30** | 8(22.2) | 0(0.0) | 1(7.1) |
| **31 – 50** | 22(61.1) | 1(16.7) | 7(50.0) |
| **>= 51** | 6(16.7) | 5(83.3) | 6(42.9) |
| **Median age (IQR)** | 36.0(31.0-45.25) | 57.5(52.5-62.5) | 47.0(39.5-55.8) |
| **Min** | 24 | 37 | 30 |
| **Max** | 70 | 65 | 59 |
| **Sex** |  |  |  |
| **Male** | 8(22.2) | 6(100.0) | 8(57.1) |
| **Female** | 28(77.8) | 0(0.0) | 6(42.9) |
| **Current role of the study participants** | | | |
| ***CIME ^†^CHW** | 36(100) | - | - |
| **Community leader** | - | 6(100.0) |  |
| **Village head** |  | 4(66.7) |  |
| **Ten-household leader** |  | 2(33.3) |  |
| **Health stakeholder (Total)** | |  | 14(100.0) |
| **Health stakeholders at community level** | |  | 6(42.9) |
| **Health assistant** |  |  | 3(50.0) |
| **Midwife** |  |  | 3(50.0) |
| ***Health stakeholders at township level*** | |  | 4(28.6) |
| **Township health assistant** |  |  | 2(50.0) |
| **Malaria supervisor** |  |  | 1(25.0) |
| **Malaria investigator** |  |  | 1(25.0) |
| ***Health stakeholders at regional level*** | |  | 4(28.6) |
| **Malaria assistant** |  |  | 2(50.0) |
| **Malaria investigator** |  |  | 1(25.0) |
| **Lab assistant** |  |  | 1(25.0) |
| **Duration of work in current role** | |  |  |
| **< 3 years** |  | 2(33.3) | 4(28.6) |
| **3 – 10 years** |  | 2(33.3) | 6(42.9) |
| **>10 years** |  | 2(33.3) | 4(28.6) |
| **Min** |  | more than a year | 2 years |
| ***Max*** |  | 31 years | 23 years |
| **<2 months** | 14(38.9) |  |  |
| **2-3months** | 14(38.9) |  |  |
| **>3 months** | 8(22.2) |  |  |
| **Min** | 5 days |  |  |
| **Max** | nearly 4 months |  |  |
| **Total** | 36(100.0) | 6(100.0) | 14(100.0) |

*Community-delivered Integrated Malaria Elimination ^†^Community Health Worker
