# Supplementary material for: Acceptability, feasibility and fidelity of an expanded role for community health workers for malaria elimination in Myanmar: A mixed-method study
Source: PLOS Glob Public Health. 2025 Aug 13;5(8):e0004986. doi: 10.1371/journal.pgph.0004986 (PMC12349089; doi:10.1371/journal.pgph.0004986)
Supplement: S4 Table — (DOCX) [file pgph.0004986.s010.docx]

S4 Table: Background characteristics of the surveyed community members

| **Characteristics** | **Hlegu (N=254)** | **Kungyangon (n=170)** | **Taikkyi (n=219)** | **Total (N=643)** |
| --- | --- | --- | --- | --- |
|  | n (%) | n (%) | n (%) | n (%) |
| **Median (IQR) age in years** | 38 (29-51) | 37 (32-53) | 40 (30-51) | 38 (29-52) |
| **Age group** |  |  |  |  |
| **18 – 34 years** | 97 (38.2) | 57 (33.5) | 78 (35.6) | 232 (36.1) |
| **35 – 65 years** | 143 (56.3) | 99 (58.2) | 128 (58.5) | 370 (57.5) |
| **>65 years** | 14 (5.5) | 14 (8.2) | 13 (5.9) | 41 (6.4) |
| **Sex** |  |  |  |  |
| **Male** | 112 (44.1) | 83 (48.8) | 97 (44.3) | 292 (45.4) |
| **Female** | 142 (55.9) | 87 (51.2) | 122 (55.7) | 351 (54.6) |
| **Residency status** |  |  |  |  |
| **Village residence** | 235 (92.5) | 169 (99.4) | 218 (99.5) | 622 (96.7) |
| **Mobile & migrant population:** | 19 (7.5) | 1 (0.6) | 1 (0.5) | 21 (3.3) |
| **Highest education level attained** | | | | |
| **No formal education** | 2 (0.8) | 0 (0.0) | 1 (0.5) | 3 (0.5) |
| **Can read and write** | 11 (4.3) | 3 (1.8) | 7 (3.2) | 21 (3.3) |
| **Primary school** | 81 (31.9) | 53 (31.2) | 79 (36.1) | 213 (33.1) |
| **Middle school** | 80 (31.5) | 50 (29.4) | 71 (32.4) | 201 (31.3) |
| **High school** | 54 (21.3) | 35 (20.6) | 43 (19.6) | 132 (20.5) |
| **University or higher** | 26 (10.2) | 29 (17.1) | 18 (8.2) | 73 (11.4) |
| **Occupation** | | | | |
| **Agriculture and livestock** | 85 (33.5) | 76 (44.7) | 117 (53.4) | 278 (43.2) |
| **Dependent** | 39 (15.4) | 30 (17.7) | 31 (14.2) | 100 (15.6) |
| **Labourer** | 40 (15.8) | 15 (8.8) | 22 (10.1) | 77 (12.0) |
| **Shopkeeper** | 36 (14.2) | 13 (7.7) | 25 (11.4) | 74 (11.5) |
| **Industry and construction** | 23 (9.1) | 14 (8.2) | 5 (2.3) | 42 (6.5) |
| **Skilled staff** | 20 (7.9) | 10 (5.9) | 8 (3.7) | 38 (5.9) |
| **^*^Other** | 11 (4.3) | 12 (7.1) | 11 (5.0) | 34 (5.3) |
| **Number of family members in the household** | | | | |
| **Range** | 1 - 9 | 1 - 9 | 1 - 10 | 1 - 10 |
| **Median (IQR)** | 4 (3-5) | 4 (3-5) | 4 (3-5) | 4 (3-5) |
| **Relationship with the household head** | | | | |
| **Self** | 106 (41.7) | 63 (37.1) | 92 (42) | 261 (40.6) |
| **Spouse** | 94 (37) | 55 (32.4) | 75 (34.3) | 224 (34.8) |
| **Son/Daughter** | 37 (14.6) | 37 (21.8) | 44 (20.1) | 118 (18.4) |
| **Parent** | 8 (3.2) | 9 (5.3) | 4 (1.8) | 21 (3.3) |
| **^†^Others** | 9 (3.5) | 6 (3.5) | 4 (1.8) | 19 (3) |
| **Presence of under-five children in the family** | | | | |
| **Yes** | 93 (36.6) | 44 (25.9) | 72 (32.9) | 209 (32.5) |
| **No** | 161 (63.4) | 125 (73.5) | 146 (66.7) | 432 (67.2) |
| **Missing** | 0 (0) | 1 (0.6) | 1 (0.5) | 2 (0.3) |
| **If present, number of under-five children in the family** | | | | |
| **Range** | 1 - 3 | 1 - 2 | 1 - 9 | 1 - 9 |
| **Median (IQR)** | 1 (1-1) | 1 (1-1) | 1 (1-1) | 1 (1-1) |

^*^Other kind of occupations included public service personnel, civil servant, nurse, waiters, pensioner, mechanic, fortune-teller, parson, administrator, barber, organist, altruist and jobless.

^†^Other relationships with household heads included siblings, uncle, nephew, niece, granddaughter, and in-laws
