# Supplementary material for: Acceptability, feasibility and fidelity of an expanded role for community health workers for malaria elimination in Myanmar: A mixed-method study
Source: PLOS Glob Public Health. 2025 Aug 13;5(8):e0004986. doi: 10.1371/journal.pgph.0004986 (PMC12349089; doi:10.1371/journal.pgph.0004986)
Supplement: S5 Table — (DOCX) [file pgph.0004986.s011.docx]

**S5 Table: Referral services received by the surveyed participants or their family members**

| **Referral services** | **Hlegu**  **(N= 46)** | **Kungyangon**  **(N= 45)** | **Taikkyi**  **(N= 51)** | **Total**  **(N= 142)** |
| --- | --- | --- | --- | --- |
|  | n (%) | n (%) | n (%) | n (%) |
| **Reasons of referral** | | | | |
| **Malaria** | 2 (4.3) | 3 (6.7) | 1 (2.0) | 6 (4.2) |
| **Dengue** | 2 (4.3) | 2 (4.4) | 11 (21.6) | 15 (10.6) |
| **Childhood diarrhoea** | 1 (2.2) | 5 (11.1) | 1 (2.0) | 7 (4.9) |
| **RDT-negative fever** | 8 (17.4) | 12 (26.7) | 14 (27.5) | 34 (23.9) |
| **Tuberculosis** | 4 (8.7) | 1 (2.2) | 5 (9.8) | 10 (7) |
| **Other causes^*^** | 28 (60.9) | 23 (51.1) | 21 (41.2) | 72 (50.7) |
| **Missing** | 2 (4.3) | 4 (8.9) | 1 (2.0) | 7 (4.9) |
| **Respondents’ satisfaction with referral services provided by the CIME CHW^#^** | | | | |
| **Satisfied** | 44 (95.7) | 44 (97.8) | 50 (98.0) | 138 (97.2) |
| **Not satisfied** | 2 (4.4) | 0 (0.0) | 1 (2.0) | 3 (2.1) |
| **Missing** | 0 (0.0) | 1 (2.2) | 0 (0.0) | 1 (0.7) |
| **Received financial support from the CIME CHW** | | | | |
| **Received** | 19 (41.3) | 11 (24.4) | 12 (23.5) | 42 (29.6) |
| **Not received** | 27 (58.7) | 34 (75.6) | 39 (76.5) | 100 (70.4) |
|  | **Hlegu**  **(n=19)** | **Kungyangon**  **(n=11)** | **Taikkyi**  **(n=12)** | **Total**  **(n=42)** |
| **Source of financial support** | | | | |
| **CIME project**  **(3000 MMK)** | 4 (21.1) | 5 (45.5) | 3 (25.0) | 12 (28.6) |
| **Other organisations (median – 14 000 MMK)** | 12 (63.2) | 4 (36.4) | 5 (41.7) | 21 (50.0) |
| **Missing** | 3 (15.8) | 2 (18.2) | 4 (33.3) | 9 (21.4) |
| **Financial support in ^†^MMK** | **Hlegu**  **(n=16)** | **Kungyangon (n=9)** | **Taikkyi**  **(n =8)** | **Total**  **(n =33)** |
| **Range** | 3,000 - 100,000 | 3,000 – 30,000 | 3,000 – 30,000 | 3,000 - 100,000 |
| **Median (IQR)** | 7 000 (4,000 - 24,000) | 3 000 (3,000-5,000) | 9,500 (17,000) | 5,000 (17,000) |

^*^Other causes of referral include cough, sneezing, dizziness, illness, for injection, cycle accident, injuries (broken hand), hepatitis, hepatic infection, stroke, arthritis, tuberculosis of central nervous system, not enough drug and deliver the patient to the health facilities by the volunteer with no mentioned reason.

^#^Community-delivered Integrated Malaria Elimination Community Health Workers

^†^1 USD = 2100 Myanmar Kyat (MMK) at the time of survey (REF: Central Bank of Myanmar)
