# Supplementary material for: Acceptability, feasibility and fidelity of an expanded role for community health workers for malaria elimination in Myanmar: A mixed-method study
Source: PLOS Glob Public Health. 2025 Aug 13;5(8):e0004986. doi: 10.1371/journal.pgph.0004986 (PMC12349089; doi:10.1371/journal.pgph.0004986)
Supplement: S6 Table — (DOCX) [file pgph.0004986.s012.docx]

S6 Table: Clinic setting of CIME^*^ community health workers (Supervision)

| **CIME clinic setting** | **Hlegu** | **Kungyangon** | **Taikkyi** | **Total** |
| --- | --- | --- | --- | --- |
|  | **(N**^†^**=29)** | **(N=17)** | **(N=23)** | **(N=69)** |
|  | n (%) | n (%) | n (%) | n (%) |
| **CIME signboard** | | | | |
| **Present** | 29(100) | 17(100) | 23(100) | 69(100) |
| **Absent** | 0(0) | 0(0) | 0(0) | 0(0) |
| **CIME signboard put at a place visible to public** | | | | |
| **Yes** | 28(96.6) | 17(100) | 22(95.7) | 67(97.1) |
| **No** | 1(3.5) | 0(0) | 1(4.4) | 2(2.9) |
| **Adequate ventilation at the place of patient care** | | | | |
| **Yes** | 27(93.1) | 17(100) | 23(100) | 67(97.1) |
| **No** | 2(6.9) | 0(0) | 0(0) | 2(2.9) |
| **Adequate lighting at the place of patient care** | | | | |
| **Yes** | 20(69.0) | 10(58.8) | 19(82.6) | 49(71.0) |
| **No** | 9(31.0) | 7(41.2) | 4(17.4) | 20(29.0) |
| **Adequate privacy in the place of patient care** | | | | |
| **Yes** | 23(79.3) | 17(100) | 17(73.9) | 57(82.6) |
| **No** | 6(20.7) | 0(0) | 6(26.1) | 12(17.4) |

^*^Community-delivered Integrated Malaria Elimination; ^†^Number of community health workers
