# Supplementary material for: Acceptability, feasibility and fidelity of an expanded role for community health workers for malaria elimination in Myanmar: A mixed-method study
Source: PLOS Glob Public Health. 2025 Aug 13;5(8):e0004986. doi: 10.1371/journal.pgph.0004986 (PMC12349089; doi:10.1371/journal.pgph.0004986)
Supplement: S7 Table — (DOCX) [file pgph.0004986.s013.docx]

S7 Table: Reviewing records and registers used by the CIME^*^ CHWs within two weeks before supervision visit

| **Recording and reporting** | **Hlegu**  **(N**^†^**=29)** | **Kungyangon**  **(N=17)** | **Taikkyi**  **(N=23)** | **Total**  **(N=69)** |
| --- | --- | --- | --- | --- |
|  | n (%) | n (%) | n (%) | n (%) |
| **Filling data in Carbonless Malaria Register** | | | | |
| **Complete** | 28(96.6) | 17(100) | 23(100) | 68(98.6) |
| **Partial** | 1(3.5) | 0(0) | 0(0) | 1(1.5) |
| **Filling data in Integrated Community Malaria Volunteer Daily Register** | | | | |
| **Complete** | 27(93.1) | 15(88.3) | 19(82.6) | 61(88.4) |
| **Partial** | 2(6.9) | 2(11.8) | 4(17.4) | 8(11.6) |
| **Filling data in CIME Record Book** | | | | |
| **Complete** | 24(82.8) | 7(41.2) | 21(91.3) | 52(75.4) |
| **Partial** | 5(17.2) | 10(58.8) | 2(8.7) | 17(24.6) |
| **All record books are filled correctly** | | | | |
| **Yes** | 25(86.2) | 16(94.1) | 22(95.7) | 63(91.3) |
| **No** | 1(3.5) | 1(5.9) | 1(4.4) | 3(4.3) |
| **Missing** | 3(10.3) | 0(0) | 0(0) | 3(4.3) |
| **Submit reports regularly** | | | | |
| **Yes** | 26(89.7) | 16(94.1) | 22(95.7) | 64(92.8) |
| **No** | 0(0) | 1(5.9) | 0(0) | 1(1.5) |
| **Missing** | 3(10.3) | 0(0) | 1(4.35) | 4(5.8) |
| **Experienced difficulties with recording and reporting** | | | | |
| **Yes** | 5(17.2) | 2(11.8) | 2(8.7) | 9(13.0) |
| **No** | 23(79.3) | 15(88.2) | 20(87.0) | 58(84.1) |
| **Missing** | 1(3.5) | 0(0) | 1(4.4) | 2(2.9) |

^*^Community-delivered Integrated Malaria Elimination Community Health Worker; ^†^number of community health workers
