# Supplementary material for: Acceptability, feasibility and fidelity of an expanded role for community health workers for malaria elimination in Myanmar: A mixed-method study
Source: PLOS Glob Public Health. 2025 Aug 13;5(8):e0004986. doi: 10.1371/journal.pgph.0004986 (PMC12349089; doi:10.1371/journal.pgph.0004986)
Supplement: S8 Table — (DOCX) [file pgph.0004986.s014.docx]

S8 Table: Stock out of medicines and commodities within two weeks before supervision visit

| **Stock out materials** | **Hlegu**  **(N^*^=29)** | **Kungyangon**  **(N=17)** | **Taikkyi**  **(N=23)** | **Total**  **(N=69)** |
| --- | --- | --- | --- | --- |
|  | n (%) | n (%) | n (%) | n (%) |
| **Malaria rapid diagnostic test** |  |  |  |  |
| **Yes** | 1(3.5) | 3(17.7) | 1(4.35) | 5(7.3) |
| **No** | 20(68.97) | 10(58.8) | 19(82.6) | 49(71.0) |
| **Missing** | 8(27.6) | 4(23.5) | 3(13.0) | 15(21.7) |
| **Paracetamol tablet** |  |  |  |  |
| **Yes** | 3(10.3) | 2(11.8) | 3(13.04) | 8(11.6) |
| **No** | 18(62.1) | 9(52.9) | 16(69.6) | 43(62.3) |
| **Missing** | 8(27.6) | 6(35.3) | 4(17.4) | 18(26.1) |
| **Multivitamin tablet** |  |  |  |  |
| **Yes** | 7(24.1) | 0(0) | 4(17.4) | 11(15.9) |
| **No** | 8(27.6) | 3(17.7) | 5(21.7) | 16(23.2) |
| **Not applicable** | 6(20.7) | 7(41.2) | 7(30.4) | 20(29.0) |
| **Missing** | 8(27.6) | 7(41.2) | 7(30.4) | 22(31.9) |
| **Zinc tablet** |  |  |  |  |
| **Yes** | 3(10.3) | 2(11.8) | 3(13.0) | 8(11.6) |
| **No** | 15(51.72) | 10(58.82) | 14(60.9) | 39(56.5) |
| **Missing** | 11(37.9) | 5(29.4) | 6(26.1) | 22(31.9) |
| **Oral rehydration solution sachet** |  |  |  |  |
| **Yes** | 2(6.9) | 2(11.8) | 4(17.4) | 8(11.6) |
| **No** | 15(51.7) | 10(58.8) | 11(47.8) | 36(52.2) |
| **Missing** | 12(41.4) | 5(29.4) | 7(30.4) | 25(36.2) |

^*^Number of community health workers
