# Supplementary material for: Acceptability, feasibility and fidelity of an expanded role for community health workers for malaria elimination in Myanmar: A mixed-method study
Source: PLOS Glob Public Health. 2025 Aug 13;5(8):e0004986. doi: 10.1371/journal.pgph.0004986 (PMC12349089; doi:10.1371/journal.pgph.0004986)
Supplement: S9 Table — (DOCX) [file pgph.0004986.s015.docx]

S9 Table: Storage of medicines and commodities among the community health workers (Supervision)

| **Storage** | **Hlegu** | **Kungyangon** | **Taikkyi** | **Total** |
| --- | --- | --- | --- | --- |
|  | **(^*^N=29)** | **(N=17)** | **(N=23)** | **(N=69)** |
|  | n (%) | n (%) | n (%) | n (%) |
| **Store properly in a safe place (box, bag)** |  |  |  |  |
| **Yes** | 27(93.1) | 17(100) | 23(100) | 67(97.1) |
| **No** | 1(3.5) | 0(0) | 0(0) | 1(1.5) |
| **Missing** | 1(3.5) | 0(0) | 0(0) | 1(1.5) |
| **Having enough space to store the stocks (shelf, box)** | | | | |
| **Yes** | 26(89.7) | 17(100) | 23(100) | 66(95.7) |
| **No** | 2(6.9) | 0(0) | 0(0) | 2(2.9) |
| **Missing** | 1(3.5) | 0(0) | 0(0) | 1(1.5) |
| **Stocks are kept far from heat, sunlight, rain/humidity** | | | | |
| **Yes** | 26(89.7) | 17(100) | 23(100) | 66(95.7) |
| **No** | 2(6.9) | 0(0) | 0(0) | 2(2.9) |
| **Missing** | 1(3.5) | 0(0) | 0(0) | 1(1.5) |
| **Having damaged or color changed rapid diagnostic test kits and medicines** | | | | |
| **Yes** | 5(17.2) | 0(0) | 4(17.4) | 9(13.0) |
| **No** | 22(75.9) | 17(100) | 19(82.6) | 58(84.1) |
| **Missing** | 2(6.9) | 0(0) | 0(0) | 2(2.9) |
| **Lancet and other blood contaminated materials are disposed into the safety box properly** | | | | |
| **Yes** | 28(96.6) | 17(100) | 22(95.7) | 67(97.1) |
| **No** | 0(0) | 0(0) | 1(4.4) | 1(1.5) |
| **Missing** | 1(3.5) | 0(0) | 0(0) | 1(1.5) |

^*^Number of community health workers
