# Supplementary material for: Acceptability, feasibility and fidelity of an expanded role for community health workers for malaria elimination in Myanmar: A mixed-method study
Source: PLOS Glob Public Health. 2025 Aug 13;5(8):e0004986. doi: 10.1371/journal.pgph.0004986 (PMC12349089; doi:10.1371/journal.pgph.0004986)
Supplement: S1 — (PDF) [file pgph.0004986.s016.pdf]

Questionnaire for cross-sectional survey

No.	Question	Answer	
Section 1: General background information and sociodemographic characteristics			
1.1	Name of interviewer		
1.2	Date of interview (dd/mm/yyyy)		
1.3	Start time (hr/min)		
1.4	End time (hr/min)		
1.5	Township name	1	Kungyangon
		2	Taikkyi
		3	Hlegu
1.6	Village name		
1.7	Age (in completed years)		
1.8	Sex	1. Male<br>2. Female	
1.9	Highest education level attained	1. No formal education<br>2. Can read and write<br>2. Primary school<br>3. Middle school<br>4. High school<br>5. University or higher	
1.10	Residency status	1. Village residence<br>2. Mobile and migrant	
1.11	Occupation	1. Rubber plantation<br>2. Farming<br>3. Gardening<br>4. Construction<br>5. Forest related jobs<br>6. Fishery<br>7. Casual Labour<br>8. Industrial worker	

		9. Other (Specify)	
1.12	Total number of family members		
1.13	Number of children under 5 years old in the family		
1.14	Relationship with household head	1	Self
		2	Wife/Husband
		3	Parent
		4	Son/Daughter
		5	Other (Specify)
Section 2: Background information of CIME volunteer			
Ko/Ma………. had attended the training at the township health department and worked as a volunteer in your village. I would like to ask you something about the services that the volunteer was providing in your village.			
2.1	Can you mention the diseases that the CIME volunteer is providing services in your village? (Select all that apply)	1. Malaria<br>2. Dengue<br>3. Tuberculosis<br>4. Childhood diarrhea<br>5. RDT-negative fever<br>6. Others (specify)	
2.2	Please rank the services in order of your favour.<br>(1 being most favourite)	1.<br>2.<br>3.<br>4.<br>5.<br>6.	
2.3	Did you receive any updated information on childhood diarrhoea service and other fever service of the CIME volunteer from your community leader?	1. Yes<br>2. No	

Section 3: Acceptability of the CIME model by community		
I would like to tell/explain some statements about the CIME volunteer providing health services that I mentioned above. If you agree with the statements, you can choose agree. If you don't, you can choose disagree. And if you don't know (or) you don't want to give any comments (or) you don't know how to say, you can choose neutral (or) don't know.		
3.1	I am satisfied with the type of services currently provided by the CIME volunteer.	1. Disagree<br>2. Neutral (or) Don't Know<br>3. Agree
3.2	I am satisfied that the CIME volunteer is skilful in giving health services.	1. Disagree<br>2. Neutral (or) Don't Know<br>3. Agree
3.3	In my opinion, the services currently provided by the CIME volunteer meet the need of community.	1. Disagree<br>2. Neutral (or) Don't Know<br>3. Agree
3.4	It is convenient for us to have a CIME volunteer in our village.	1. Disagree<br>2. Neutral (or) Don't Know<br>3. Agree
3.5	I am satisfied with the way the CIME volunteer is communicating with the community.	1. Disagree<br>2. Neutral (or) Don't Know<br>3. Agree
3.6	I am satisfied that the CIME volunteer is giving health services with compassion.	1. Disagree<br>2. Neutral (or) Don't Know<br>3. Agree
3.7	I am satisfied with the childhood diarrhoea services and other fever services from the CIME volunteer.	1. Disagree<br>2. Neutral (or) Don't Know<br>3. Agree
3.8	I accept and am willing to get the same type of services from the CIME volunteers in the future.	1. Disagree<br>2. Neutral (or) Don't Know<br>3. Agree

Section 4: Fidelity of the CIME volunteers		
4.1	Have you or your family member ever received any health services from the CIME volunteer within the last 7 months?	1. Yes<br>2. No (SKIP to 4.2)
4.1.1.	If yes, how many times did you visit the CIME volunteer during the last 7 months?	…………………………
4.1.2	What health services have you or your family member received from the CIME volunteer during the last 7 months?<br>(Select all that apply)	1. Malaria<br>2. Dengue<br>3. Tuberculosis<br>4. Childhood diarrhea<br>5. RDT-negative fever<br>6. Other (specify)
4.2	Have you received any health education/ information from the CIME volunteer within the last 7 months?	1. Yes<br>2. No (SKIP to 4.3)
4.2.1	If yes, for what diseases have you received health education/information from the CIME volunteer? (Select all that apply)	1. Malaria<br>2. Dengue<br>3. Tuberculosis<br>4. Childhood diarrhea<br>5. RDT-negative fever<br>6. Other (specify)
4.2.2	Do you think health education/information received from the CIME volunteer was useful to you?	1. Yes (SKIP to 4.3)<br>2. No
4.2.3	If you do not think it is useful, please mention why.	
4.3	Did the CIME volunteer visit your house for larva control activities within the last 7 months?	1. Yes<br>2. No (SKIP to 4.4)
4.3.1	If yes, when was the last time the CIME volunteer visited your house for larva control within 7 months?	

4.4	Have you or your family member been referred by the CIME volunteer to a health facility within the last 7 months?	1. Yes<br>2. No (SKIP to 4.5)
4.4.1	If yes, what was the reason for referral?	
4.4.2	Did you receive any financial support to enable you to attend a health facility following referral?	1. Yes<br>2. No (SKIP to 4.4.4)
4.4.3	If yes, how much did you receive from the CIME volunteer?	
4.4.4	Were you satisfied with the referral service of the CIME volunteer?	1. Yes (SKIP to 4.5)<br>2. No
4.4.5	If you were not satisfied with the referral service by the CIME volunteer, please mention why.	
4.5	What type of disease services do you want to receive from the CIME volunteer in future? (eg. diarrhoea/ contraception/ other)	

4.6	What type of health services do you want to receive from the CIME volunteer in future?	1	Health Education service
		2	Disease prevention service
		3	Disease Diagnosis service
		4	Treatment service
		5	Referral service
		6	Other (Please mention)

We would like to top up your phone with bill 3000 MMK in appreciation of your participation in this survey. Can you tell me the phone number to which you want to receive phone bill?	09-

(END) Thank you for your time and participation.
