# Supplementary material for: Acceptability, feasibility and fidelity of an expanded role for community health workers for malaria elimination in Myanmar: A mixed-method study
Source: PLOS Glob Public Health. 2025 Aug 13;5(8):e0004986. doi: 10.1371/journal.pgph.0004986 (PMC12349089; doi:10.1371/journal.pgph.0004986)
Supplement: S2 — (PDF) [file pgph.0004986.s017.pdf]

Semi-structured interview guides

Interview Guide for In-depth Interviews with the Health Stakeholders

This is the interview guide for the in-depth interview with the health stakeholders managing the CIME Volunteer activities (NMCP staff and BHSs of the CIME Volunteers' village). This interview is expected to be conducted in about 1.5 hours.

**Information about the interview session**		
1.1.	Name of the interviewer	
1.2.	Date (dd/mm/yyyy)	
1.3.	Start time	
1.4.	End time	
1.5.	Archival code	

**Is it OK to audio-record this conversion? (Yes/No)**	

**Background information of the participant**		
2.1.	Age	
2.2.	Sex	
2.3.	State/Region	
2.4.	Department (NMCP, Sub-/RHC)	
2.5.	Could you briefly describe your current designation?	

2.6.	How long have you been working in the current position?	
2.7.	What are your roles and responsibilities relating the community-based health volunteers/workers?	

**CIME Volunteer Activities and Supervision by the Health Staff**	
3.1.	How many community-based health volunteers/workers do you have in your area?<br><br>a. How many of them have worked as CIME Volunteers?
3.2.	Do you usually conduct monitoring and supervision activities/visits to your CIME volunteers? (during field visits/ monthly meetings)<br><br>a. How frequently do you conduct monitoring and supervision activities? How many intervals do you have between the two monitoring and supervision activities/visits?<br>b. What do you usually do during monitoring and supervision activities/visits?<br>i. On-site assessment and training<br>ii. Checking their performance in service provision<br>iii. Check record and reports<br>iv. Checking stock management<br>c. What significant findings have you found during monitoring and supervision activities/visits?<br>i. Do the CIME Volunteers follow according to the guidelines?<br>ii. What are the common mistakes that the CIME Volunteers usually make?<br>d. What were your actions on these findings?

**Barriers and Enablers of CIME Volunteer Activities**	
4.1.	**Challenges**

	a. What are the challenges <u>encountered by the CIME Volunteers</u> during their service provision (including challenges due to covid 19 pandemic situation and security issues)? How do you solve them?<br>i. Malaria<br>ii. Dengue<br>iii. TB<br>iv. Childhood diarrhoea<br>v. Fever<br>vi. Referral<br>vii. BCC<br>b. What are the challenges <u>encountered by the health staff</u> (NMCP staff or BHSs) relating to the CIME Volunteers? How do you solve them?<br>i. Referral<br>ii. Records and reports<br>iii. Monitoring and supervision<br>iv. Cooperation<br>c. What are the challenges <u>encountered by the respective disease control program</u>s (VBDC especially NMCP, NTP, Child health, etc.) relating to the CIME Volunteers? How do you solve them?<br>i. Are there any drop-outs of the CIME Volunteers? How many of them are there? What are the reasons of the dropouts? How do you solve that problem?<br>d. Are these challenges the same as for ICMVs and CIME Volunteers? What are the new challenges arising with the CIME Volunteers? (Or) What are the weak points of CIME model compared to the ICMV model? Why do these new challenges arise?
4.2.	What are the strengths of the CIME model favouring it over the ICMV model?<br>a. At volunteer level<br>b. At community level<br>c. At supervisor level (NMCP staff and BHSs)

	d. At programmatic level
4.3.	How can the CIME model be improved to overcome these challenges?<br><br>a. At volunteer level<br>b. At community level<br>c. At supervisor level (NMCP staff and BHSs)<br>d. At programmatic level

**Perspectives on CIME Volunteer Activities by Health Stakeholders**	
5.1.	How do you find the service provision of CIME Volunteers? How do you think about the **service quality** of the CIME Volunteers?<br><br>a. Do you think the CIME Volunteers are competent with the tasks they are supposed to do? (Are they reliable?) Why?<br>b. Do you think the CIME Volunteers are fully equipped with all necessary drugs and equipment for their services? Why?<br>c. Do you think the CIME Volunteers perform their tasks in a timely manner? (Recording, Reporting, Case notification, etc.) If not, what do you think are the reasons?<br>d. How do you find communication of the CIME Volunteers with you (their supervisors)? Why?<br>e. Do the CIME Volunteers cooperate well with you (their supervisors)? What the common occasions that you and the CIME Volunteers can/cannot cooperate well?<br>f. How do you find the attitude of the CIME Volunteer with their job? and with you (their supervisors)? Discuss about it. Why?
5.2.	What do you think about the diseases omitted from the model while transforming from ICMV to CIME model? (**Filariasis, Leprosy, HIV/STI**)<br><br>a. How does it have an impact on your program (or) health implementation services?

	- In disease control activities and health services provided by basic health staff<br>- In the community<br>b. Is it acceptable for you if these diseases will not be handled anymore by all community-based health workers in the future? Why?
5.3.	What do you think about the new diseases/tasks added to the model while transforming from ICMV to CIME model? (**Childhood diarrhea, RDT-negative fever, Malaria surveillance activities**)<br><br>a. How does it have an impact on your program (or) supervisors/ basic health staffs' health implementation services/disease control activities and community?<br>b. Is it possible/acceptable for you if these diseases/conditions will be included in community-based health worker models in the future? Which diseases/tasks that added to the model would be acceptable? Why?
5.4.	How do you think about recording and reporting conducted by the CIME Volunteers?<br><br>a. How does it have an impact on your program (or) supervisors/ basic health staffs' health implementation services/disease control activities?<br>b. Are you satisfied with them? Why?
5.5.	How do you find your management (especially monitoring and supervision) with the CIME Volunteers?<br><br>a. How does it have an impact on your program (or) supervisors/ basic health staffs' health implementation services/disease control activities?<br>b. Are you satisfied with them? Why?
5.6.	(**As a BHS**) How do you think regarding the patient referral service provided by the CIME Volunteers?<br><br>c. How does it have an impact on your program (or) supervisors/ basic health staffs' health implementation services/disease control activities, and community?<br>a. Are you satisfied with them? Why?

Perspectives on the CIME model training (Experience, opinions and suggestions)	
6.1.	Relating to the CIME model training,<br><br>• Is there anything that you like?<br>• Is there anything that you dislike/ you would like to improve to be better? Is there any difficulty in attending the CIME model training?<br>- Training Contents<br>- Trainers<br>- Teaching technique/method<br>- Training supports (Venue, Video Display, Refreshments, Daily Perdium & Travel Support, etc.)
6.2	How should the CIME model training be conducted to be better?

Overall	
7.1.	As a health stakeholder, are you satisfied with the overall performance of the CIME Volunteers?
7.2.	What are the significant impacts of switching from the ICMV model to the CIME model?<br><br>a. At volunteer level<br>b. At community level<br>c. At supervisor level (NMCP staff and BHSs)<br>d. At programmatic level
7.3.	Which do you prefer? ICMV or CIME? Why?
7.4.	Do you think all other ICMVs should also be switched into CIME Volunteers? Why?

	a. Is it possible in the current setting? Why?<br>b. What should we do if we want to do so?

**Conclusion**	
	Do you have any questions for me?<br><br>Thank you very much for your participation.

**End of session**

Interview Guide for Key Informant Interview with the Village Leaders

This is the interview guide for the key informant interview with the village leaders (mostly probably Village Administrators) of the CIME Volunteers' villages. This interview is expected to be conducted in about 1.5 hours.

**Information about the interview session**		
1.1.	Name of the interviewer	
1.2.	Date (dd/mm/yyyy)	
1.3.	Start time	
1.4.	End time	
1.5.	Archival code	

**Is it OK to audio-record this conversion? (Yes/No)**	

**Background information of the participant**		
2.1.	Age	
2.2.	Sex	
2.3.	State/Region	
2.4.	How long have you been living in this village? Were you born here?	
2.5.	How long have you been working as a Village Administrator (or a village leader) in this village?	

CIME Volunteer activities in the village	
3.1.	Have you noticed any volunteers in your village?
3.2.	If yes,<br><br>• Could you please list these volunteers what they are? (types of volunteers)<br>• What kind of disease services did they provide? (e.g malaria,TB,nutrition,etc.)
3.3.	Do you know the Community-delivered Integrated Malaria Elimination Volunteer, or the CIME Volunteer, in your village?
3.4.	Could you describe the CIME Volunteer? (Who they are, what they do, etc.)<br><br>Do you know that the Integrated Community-based Malaria Volunteer (ICMV) has been transformed into a CIME Volunteer?
3.5.	What services are given by the CIME Volunteer? Can you please describe these services in detail? How is he/she doing them?<br><br>a. Malaria<br>b. Dengue<br>c. Tuberculosis<br>d. Childhood diarrhoea<br>e. Fever<br>f. Referral<br>g. BCC

Perspectives on the CIME Volunteer Services	
4.1.	Have you or your family ever **visited the CIME Volunteer**? Or have you ever seen or heard of your village people visited the CIME Volunteer?<br><br>a. How many times/ How frequently have you encountered such occasions?<br>b. Why did you or they visited the CIME Volunteer?

	c. What services did you or they received form the CIME Volunteer? Could you please describe these services in detail?<br>d. How were the outcomes of these services? Why? How do you think?<br>e. Are you satisfied with these services of the CIME Volunteer? Why?<br>f. Do you think your village people are satisfied with these services of the CIME Volunteer? Why?
4.2.	Have you ever seen, heard, or informed about any **health education (BCC) activities** by the CIME Volunteer? Have you ever attended these sessions by yourself?<br><br>a. How many times? Or how frequently?<br>b. Did he/she need your help in arranging such activities? If so, for what and how did you help him/her?<br>c. What are the topics discussed by the CIME Volunteer during these sessions?<br>d. How do you find these health education activities of the CIME Volunteer? Are they understandable? How do these activities benefit your village people?<br>e. Are you satisfied with these services of the CIME Volunteer? Why?<br>f. Do you think your village people are satisfied with these services of the CIME Volunteer? Why?
4.3.	Have you ever seen, heard, or informed about any **larva control activities** by the CIME Volunteer?<br><br>a. How many times? Or how frequently?<br>b. Did he/she need your help in arranging for such activities? If so, for what and how did you help him/her?<br>c. What does the volunteer do during these activities? Could you please describe them in detail?<br>d. How do you find these larva control activities of the CIME Volunteer? How do these services benefit your village people?<br>e. Are you satisfied with these services of the CIME Volunteer? Why?<br>f. Do you think your village people are satisfied with these services of the CIME Volunteer? Why?

<table>
<tr><td>4.4.</td><td>Have you ever encountered (seen, heard, or informed about) the CIME Volunteer referring a patient to the health centers, clinics, or hospitals?

a. How many times? Or how frequently?
b. Do you know what the CIME Volunteer refers his/her patients for? Where/ Whom does he/she refer his/her patients to?
c. Have you noticed any difficulties <u>encountered by the CIME Volunteers</u> during such referrals? If so, could you please describe them? (including difficulties due to covid 19 pandemic situation, security issues)
d. Have you noticed any difficulties <u>encountered by the village people</u> during such referrals? If so, could you please describe them? (including difficulties due to covid 19 pandemic situation, security issues)
e. Did the volunteer or the village people need your help in arranging for such referrals? If so, for what and how did you help him/her?
f. How do you find these referral services of the CIME Volunteer? How do these services benefit your village people?
g. Are you satisfied with these referral services of the CIME Volunteer? Why?
h. Do you think the village people are satisfied with these referral services of the CIME Volunteer? Why?</td></tr>
<tr><td>4.5.</td><td>How do you think about the service quality of the CIME Volunteer?

a. Do you think the CIME Volunteer is competent with the tasks he/she is supposed to do? (Is he/she reliable for your village people?) Why?
b. Do you think the CIME Volunteer is fully equipped with all necessary drugs and equipment for his/her services? Why?
c. Can the village people easily get the available services from the CIME Volunteer whenever they need them? If not, why?
d. Does the CIME Volunteer willingly explained everything during service provision? How is he/she doing that?
e. How do you find the communication of the CIME Volunteer with others? Why? (Is he/she patient? Kind? Caring?)</td></tr>
</table>

	f. How do you find the attitude of the CIME Volunteer with his job? and with his clients? Why?<br>g. Do the village people need to pay for the services they received from the CIME Volunteers? If so, what do they need to pay for? And how much?
4.6.	In addition to the difficulties encountered by the CIME Volunteer and the village people during service provision (both curative and preventive services), what other challenges do your village encounter related to the CIME Volunteer?(including challenges due to the covid 19 pandemic and security issues)<br><br>a. How can these challenges be overcome?<br>b. In addition to the current CIME Volunteer services, what other services do you want to receive from the CIME Volunteer?

**Overall**	
5.1.	Considering all aspects of the CIME Volunteer and his/her services in your village,<br><br>a. Are you, as a village leader, satisfied with the overall performance of the CIME Volunteer and his/her service provision?<br>b. Do you think your village people are satisfied with the overall performance of the CIME Volunteer and his/her service provision?<br>c. How does the presence of such a CIME Volunteer benefit your village and village people?<br>d. Are you and your village people happy having such a CIME Volunteer in your village? Why?
5.2.	**Comparing** the **CIME** Volunteer services to those when he/she previously worked as an Integrated Community-based Malaria Volunteer (**ICMV**),<br><br>a. What do you think are the main differences between CIME and ICMV?<br>b. What are the strengths of having a CIME Volunteer rather than an ICMV? What are the weaknesses of a CIME Volunteer compared to an ICMV?

	c. What are the differences in the impact of the CIME and ICMV on your village and village people?<br>d. Which do you prefer? ICMV or CIME? Why?<br>e. Do you think all other ICMVs should also be switched into CIME Volunteers? Why?

**Conclusion**	
	Do you have any questions for me?<br><br>Thank you very much for your participation.

**End of session**

Focus Group Discussion Guide

Responsible persons	Responsibility
Facilitator (F)	Lead the overall process and facilitate discussion to obtain enriched data using an ethical approach
Note taker (NT)	Note-taking, audio recording and supplementary facilitation
Translator	Translation of facilitator and participants discussion where necessary.

**Information about the discussion session**		
1.1.	Name of the facilitator	
1.2.	Name of the notetaker	
1.3.	Name of the translator	
1.4.	Date (dd/mm/yyyy)	
1.5.	Start time	
1.6.	End time	
1.7.	Archival code	

**Is it OK to audio-record this conversion? (Yes/No)**	

**Background information of the participant**		
2.1.	Sex of participants (group)	

2.2.	Age range of participants	
2.3.	Location (State/Region)	
2.4.	How many years of work experience as an ICMV?	
2.5.	How long have you worked in your current position, as a CIME volunteer (in months)?	
2.6.	What kind of health care services do you provide as a CIME volunteer in your working village/area?	

**Malaria related services**	
3.1.	Which types of services do you provide regarding malaria? How do you perform RDT testing, DOT, malaria referral, and malaria surveillance?
3.2.	Are you ok when you provide these services (RDT testing/ DOT/ referral/ malaria surveillance)?<br>(a) Do you have any difficulties when you provide these services (RDT testing/ DOT/ referral/ malaria surveillance)? What are they? How do you solve them?<br>(b) What are the facilitators when you provide these services (RDT testing/ DOT/ referral/ malaria surveillance)?
3.3.	Comparing ICMV to CIME:<br>• What has changed with respect to how you provide malaria services?<br>• What difficulties do you have when you provide malaria services?<br>• What is the strength of CIME over ICMV in increasing RDT testing and finding more malaria-positive cases?
3.4.	What is the impact of CIME on malaria in the community?

3.5.	Do you prefer CIME over ICMV related with malaria? Why?

**Dengue related services**	
4.1.	What services do you provide for dengue? How do you perform larva control in the community and referral service for dengue suspected patients?
4.2.	Are you ok to provide these services (larva control in the community/ referral service)?<br>(a) Do you have any difficulties when you provide these services (larva control in the community/ referral service)? What are they? How do you solve them?<br>(b) What are the facilitators when you provide these services (larva control in the community/ referral service)?
4.3.	In comparison between ICMV and CIME,<br>• What changes do you have when you provide dengue services?<br>• What difficulties do you have when you provide dengue services?<br>• What is the strength of CIME over ICMV in doing larva control activity by community and referring more dengue suspected patients?
4.4.	What is the impact of CIME on dengue in the community?
4.5.	Do you prefer CIME over ICMV related with dengue? Why?
**TB related services**	
5.1.	What services do you provide for TB? How do you perform referral service for TB suspected patients?
5.2.	Are you ok to provide these services (referral service and others)?<br>(a) Do you have any difficulties when you provide these services (referral service and others)? What are they? How do you solve them?

	(b) What are the facilitators when you provide these services (referral service and others)?
5.3.	In comparison between ICMV and CIME,<br>• What changes do you have when you provide TB services?<br>• What difficulties do you have when you provide TB services?<br>• What is the strength of CIME over ICMV in referring more TB suspected patients?
5.4.	What is the impact of CIME on TB in the community?
5.5.	Do you prefer CIME over ICMV related with TB? Why?

**Childhood diarrhea related services**	
6.1.	What services do you provide for childhood diarrhea? How do you perform case management and referral service for childhood diarrhoea patients?
6.2.	Are you ok to provide these services (case management/ referral service)?<br>(a) Do you have any difficulties when you provide these services (case management/ referral service)? What are they? How do you solve them?<br>(b) What are the facilitators when you provide these services (case management/ referral service)?
6.3.	Do you prefer to include childhood diarrhea service provision in CIME? Why?
6.4.	What is the impact of CIME on childhood diarrhoea in the community?

**RDT negative fever related services**	
7.1.	What services do you provide for fever patients? How do you perform referral service for RDT negative fever patients?

7.2.	Are you ok to provide these services (referral services and others)?<br><br>(a) Do you have any difficulties when you provide these services (referral services and others)? What are they? How do you solve them?<br>(b) What are the facilitators when you provide these services (referral services and others)?
7.3.	Do you prefer to include RDT negative fever service provision in CIME? Why?
7.4.	What is the impact of CIME on RDT negative fever in the community?

**Health education services**	
8.1.	How are you conducting health education sessions in the community? How many times do you provide per month? What topics do you provide?
8.2.	In comparison between ICMV and CIME,<br><br>• What changes do you have when you conduct health education sessions in the community?<br>• What difficulties do you have when you conduct health education sessions in the community? How do you solve them?<br>• What is the strength of CIME over ICMV in giving health education in the community?
8.3.	What is the impact of CIME on conducting health education sessions in the community?

**Recording & Reporting**	
9.1.	What do you do in every month relating to patient registers/records, activities registers/records, and sending monthly reports? Which kinds of registers/records & monthly reports do you record and report?

	What changes do you have when you do record and send the reports in comparison between ICMV and CIME?<br><br>In which way, do you send the monthly reports to the respective focal and health centers?
9.2	Are you ok when you conduct recording and sending the reports?<br><br>• Do you have any difficulties? What are these difficulties/barriers? How do you solve them?<br>• Is there any facilitator when you conduct them (recording and reporting)? What are these facilitators?
9.3	Is there anything related to the recording and reporting that you would like to change/improve in order to facilitate when you do them? If so, how would you like to change/ improve them?

**Current Issues/Special Situations (Experience, Opinions and Suggestions of the CIME volunteers)**	
10.1.	Is there any impact on your implementation services and activities conducted due to the current covid 19 pandemic situations?<br><br>What difficulties/barriers are you facing because of it? (as an ICMV volunteer /as an CIME volunteer)<br><br>How do you solve them?<br><br>Which kind of preparation do you think you should prepare if you must face the similar pandemic situation in the future? Which supports do you need for it?
10.2	Is there any impact on your implementation services and activities due to the current political situation?

	What difficulties/barriers are you facing because of it? (as an ICMV volunteer /as an CIME volunteer)<br><br>How do you solve them?<br><br>Which kind of preparation do you think you should prepare if you must face that kind of difficult situation? Which supports do you need for it?

**The CIME model training (Experience, opinions and suggestions of the CIME volunteers)**	
11.1.	Relating to the CIME model training,<br><br>• Is there anything that you like?<br>• Is there anything that you dislike/ you would like to improve to be better? Is there any difficulty in attending the CIME model training?<br>- Training Contents<br>- Trainers<br>- Teaching technique/method<br>- Training supports (Venue, Video Display, Refreshments, Daily Perdium & Travel Support, etc.)
11.2	How should the CIME model training be conducted to be better?

**Community participation**	
12.1.	Does the community accept the CIME model more than ICMV? If so, why?

**Overall**	
13.1.	Which one do you prefer, CIME or ICMV?<br><br>What is your opinion related to removing the ICMV diseases like filariasis, leprosy & syphilis in the CIME model? Why do you think like that?
13.2.	Do you think all ICMVs should also be transformed into the CIME Volunteers in the future?<br>Why?
13.3.	Do you want to continue as the CIME volunteer?
13.4.	Which kind of improvements are needed for CIME model to be better in the future?

**Conclusion**	
	This is the end of my questions.<br>Do you have anything else you would like to say about this research?<br>Do you have any questions for me?<br><br>Thank you very much for your participation.

**End of session**
