# Supplementary material for: Acceptability, feasibility and fidelity of an expanded role for community health workers for malaria elimination in Myanmar: A mixed-method study
Source: PLOS Glob Public Health. 2025 Aug 13;5(8):e0004986. doi: 10.1371/journal.pgph.0004986 (PMC12349089; doi:10.1371/journal.pgph.0004986)
Supplement: S3 — (PDF) [file pgph.0004986.s018.pdf]

Field Observation Checklist for Community Based Integrated Malaria Elimination (CIME) Volunteer Activities

This is the field observation checklist for CIME project research team members. The filed observation is expected to be conducted in about 2 hours.

**1.Basic information**					
1.1.	State/Region		1.2.	Township	
1.3.	Name of RHC		1.4.	Name of sub-centre	
1.5.	Village's name		1.6.	Date(D/M/Y)	
1.7.	Start time		1.8.	End time	
1.9.	Name of Observer				

**2.Village/Work information**				
2.1.	Socio-economic status			
2.2.	Geographical condition	☐ Plain<br>☐ Foothill<br>☐ Hill<br>☐ Near the field	☐ Dam/stream/river side<br>☐ Near the forest<br>☐ others	
2.3.	Road condition	☐ Earth ☐ Concrete ☐ Nylon-tar		
2.4.	Mobile phone access	☐ Yes<br>☐ No	If "Yes",<br>The connection is	☐ Good<br>☐ Poor
2.5.	Electricity	☐ Government ☐ Solar ☐ Generator ☐ Private water turbine generator<br>☐ Others		
2.6.	Presence of government health facility in the village	☐ Yes ☐ No		
2.7.	Distance to nearest health facility	With motorbike/car	.... min/hr	
		On foot	.... min/hr	
2.8.	Presence of private clinic in the village	☐ Yes<br>☐ No	If "Yes", how many clinics?	...................<br>clinics
2.9.	Presence of pharmacist shop in the village	☐ Yes<br>☐ No	If "Yes", how many clinics?	....................<br>shops

**3.Setting**			
3.1.	CIME signboard place at a visible place to public	☐ Yes ☐ No<br>☐ Not found	
3.2.	Place of examination of patient	☐ Good ventilation<br>☐ Good lighting<br>☐ Private place	

		☐ Others ....

4.Records, reports, and references			
			Reason if "not found"
4.1.	Records, report, and references	☐ Carbonless malaria register<br>☐ ICMV daily register<br>☐ CIME volunteer record book<br>☐ Referral form<br>☐ Malaria CI and classification form<br>☐ ICMV quarterly report<br>☐ Other	

**Evaluation of records and forms (Records in previous six month)**								
**Carbonless malaria register**		**Month 1**	**Month 2**	**Month 3**	**Month 4**	**Month 5**	**Month 6**	**Remark**
4.2.	Number of patients RDT tested							
4.3.	Number of P.f positive patients							
4.4.	Number of P.f positive patients provided with correct treatment							
4.5.	Number of P.v positive patients							
4.6.	Number of P.v positive patients provided with correct treatment							
4.7.	Number of Mixed patients							
4.8.	Number of Mixed patients provided with correct treatment							
4.9.	Number of malaria patients referred							
4.10.	Reason for referral	☐ Danger signs ☐ Pregnancy ☐ Infant ☐ Other						

ICMV daily register		Month 1	Month 2	Month 3	Month 4	Month 5	Month 6	Remark
4.11.	Number of patients attended							
4.12.	Number of RDT (-) ve fever patients							
4.13.	Number of childhood diarrhoea cases							
4.14.	Number of TB suspected patients							
4.15.	Number of Dengue suspected patients							
4.16.	Number of filariasis suspected patients							
4.17.	Number of leprosy suspected patients							
4.18.	Number of HIV suspected patients							
4.19.	Number of STD suspected patients							
4.20.	Number of patients referred							

CIME record book		Month 1	Month 2	Month 3	Month 4	Month 5	Month 6	Remark
4.21.	Number of HE session							
4.22.	Number of participants attended in HE session							
4.23.	Number of malaria case notifications within 24 hours							
4.24.	Number of initial case investigations and classification by CIME							
4.25.	Number of malaria patient provided DOT							

Evaluation of filling, recording, and storage of registers, forms, and difficulties			
4.26.	Records and forms are used correctly<br>☐ Carbonless malaria register	☐Yes ☐No	If "No", how the record is not used correctly?
	☐ ICMV daily register	☐Yes ☐No	
	☐ CIME record book	☐Yes ☐No	
	☐ Referral form	☐Yes ☐No	
	☐ Malaria CI and classification form	☐Yes ☐No	

	☐ ICMV quarterly report	☐Yes ☐No	
	☐ Others	☐Yes ☐No	
4.27.	How are the records reported?	☐ Volunteer sends the report<br>☐ Township focal person collects<br>☐ Send via another person<br>☐ Other	
4.28.	How frequently are the records reported?	☐ Fortnightly<br>☐ Monthly<br>☐ Every 2 months<br>☐ Every 3 months<br>☐ others<br>..........................	If not reported regularly, which records are not reported regularly?
4.29.	Registers, forms, and reference are kept in a safe place. (Separate box, not easily accessible place)	☐ Yes<br>☐ No	
4.30.	There are difficulties in recording registers.	☐ Yes<br>☐ No	If "Yes", which records are they and what are the difficulties?

<table>
<tr><th colspan="8">5. Evaluation of logistic management of medicines and commodities (to check the balance of medicines and commodities in previous six months)</th></tr>
<tr><th colspan="2">Medicines and commodities</th><th>Previous month balance</th><th>Received this month</th><th>Opening balance</th><th>Used in this month</th><th>Final balance</th><th>Expired date</th></tr>
<tr><td>5.1.</td><td>RDT</td><td></td><td></td><td></td><td></td><td></td><td></td></tr>
<tr><td>5.2.</td><td>ACT 24</td><td></td><td></td><td></td><td></td><td></td><td></td></tr>
<tr><td>5.3.</td><td>ACT 18</td><td></td><td></td><td></td><td></td><td></td><td></td></tr>
<tr><td>5.4.</td><td>ACT 12</td><td></td><td></td><td></td><td></td><td></td><td></td></tr>
<tr><td>5.5.</td><td>ACT 6</td><td></td><td></td><td></td><td></td><td></td><td></td></tr>
<tr><td>5.6.</td><td>CQ</td><td></td><td></td><td></td><td></td><td></td><td></td></tr>
<tr><td>5.7.</td><td>PQ</td><td></td><td></td><td></td><td></td><td></td><td></td></tr>
<tr><td>5.8.</td><td>Paracetamol</td><td></td><td></td><td></td><td></td><td></td><td></td></tr>
<tr><td>5.9.</td><td>Multivitamin</td><td></td><td></td><td></td><td></td><td></td><td></td></tr>
<tr><td>5.10.</td><td>Zinc</td><td></td><td></td><td></td><td></td><td></td><td></td></tr>
<tr><td>5.11.</td><td>ORS</td><td></td><td></td><td></td><td></td><td></td><td></td></tr>
<tr><td>5.12.</td><td colspan="2">There is stock out of medicines and commodities in previous six months</td><td>☐ Yes
☐ No</td><td colspan="4">If “Yes”, which were they and how long was the stock out?</td></tr>
<tr><td>5.13.</td><td colspan="2">There is possible RDT stock out next month.
(Final balance < average use in previous three months)</td><td>☐ Yes
☐ No</td><td colspan="4"></td></tr>
<tr><td>5.14.</td><td colspan="2">There is possible RDT overstock in coming months.
(Months eligible to use > duration before expiry)</td><td>☐ Yes
☐ No</td><td colspan="4">Months eligible to use = final balance ÷ average use in previous three months
Duration before expiry = expiry date – date of field observation</td></tr>
<tr><td>5.15.</td><td colspan="2">Labels of medicines are well visible.</td><td>☐ Yes
☐ No</td><td colspan="4"></td></tr>
<tr><td>5.16.</td><td colspan="2">Expired drugs are kept in separate box or place.</td><td>☐ Yes
☐ No</td><td colspan="4"></td></tr>
<tr><td>5.17.</td><td colspan="2">Volunteer can check the expiry date of medicines and commodities.</td><td>☐ Yes
☐ No</td><td colspan="4"></td></tr>
<tr><td>5.18.</td><td colspan="2">How are the expired medicines and commodities managed? (Ask the volunteer)</td><td colspan="5"></td></tr>
<tr><th colspan="3">Records of medicines and commodities</th><th></th><th colspan="4">If “No”, why?</th></tr>
<tr><td>5.19.</td><td colspan="2">There is stock record book.</td><td>☐ Yes
☐ No</td><td colspan="4"></td></tr>
</table>

5.20.	Medicines and commodities are recorded correctly.	☐ Yes<br>☐ No	
5.21.	Medicines and commodities are recorded up to date.	☐ Yes<br>☐ No	
5.22.	There is compatibility between record of medicine use and record of patients.	☐ Yes<br>☐ No	
5.23.	There is compatibility between final balance at the time of observation and ground balance.	☐ Yes<br>☐ No	If “No”, which medicine and commodities are they?
**Storage of medicine and commodities**			**If “No”, why?**
5.24.	Medicine and commodities are kept properly in a safe place. (Boxes)	☐ Yes<br>☐ No	
5.25.	There is enough storage space (Cupboard, box) to keep the medicine and commodities.	☐ Yes<br>☐ No	
5.26.	Medicines and commodities are kept away from heat, sunlight, raindrops, and humidity.	☐ Yes<br>☐ No	
5.27.	There are damage, wear and tear, and change of colour of the medicines and commodities.	☐ Yes<br>☐ No	
5.28.	Needle and blood-stained material are discarded properly into the safety box.	☐ Yes<br>☐ No	
5.29.	How is the used safety box discarded? (To ask the volunteer)		

Supported packages			
**Items**			**Current condition**
5.30.	CIME backpack, box	☐ Yes<br>☐ No	
5.31.	Thermometer	☐ Yes<br>☐ No	
5.32.	Weighing scale	☐ Yes<br>☐ No	
5.33.	Torch light	☐ Yes<br>☐ No	
5.34.	Malaria treatment guideline card	☐ Yes<br>☐ No	
5.35.	Flip chart, poster, pamphlets	☐ Yes<br>☐ No	
5.36.	Pen to record on RDTs	☐ Yes<br>☐ No	
5.37.	Ziplock bag to store RDTs	☐ Yes<br>☐ No	
5.38.	Code sticker to mark RDTs	☐ Yes<br>☐ No	

6. Evaluating the performance of the CIME volunteer		
6.	The following tasks are carried out during the field observation:	☐ Not any task<br>☐ Patient examination, recording and referral<br>☐ Malaria surveillance<br>☐ Disease control, TB DOTS assistant<br>☐ Health education

**Case management**			
**6.1.**	**Malaria**		
History taking and physical examination			
6.1.1.	Asking the patients background information<br>(Name, age, sex, pregnancy, address, occupation)	☐ Yes<br>☐ No	
6.1.2.	Assessing signs and symptoms of malaria	☐ Yes<br>☐ No	
6.1.3.	Assessing travelling history	☐ Yes<br>☐ No	
6.1.4.	Assessing danger signs	☐ Yes<br>☐ No	
6.1.5.	Measuring the body temperature correctly	☐ Yes<br>☐ No	

Malaria blood test with RDT			
6.1.6.	Checking expiry date and wear and tear of RDTs	☐ Yes<br>☐ No	
6.1.7.	Recording information of the patient on RDT cassette	☐ Yes<br>☐ No	
6.1.8.	Wearing gloves	☐ Yes<br>☐ No	
6.1.9.	Cleaning the fingertip of the patient with spirit	☐ Yes<br>☐ No	
6.1.10.	Wiping the first blood with clean cotton wool	☐ Yes<br>☐ No	
6.1.11.	Collecting blood with pipe up to the defined limit (5 μl)	☐ Yes<br>☐ No	
6.1.12.	Putting the blood correctly in “S” mark	☐ Yes<br>☐ No	
6.1.13.	Putting the four drops of buffer into the “S” mark	☐ Yes<br>☐ No	
6.1.14.	Recording time on RDT	☐ Yes	

		☐ No	
6.1.15.	Reading the blood test within 15 – 30 min	☐ Yes<br>☐ No	
6.1.16.	Explaining the result to the patient	☐ Yes<br>☐ No	
6.1.17.	Blood test result	☐ P.f ☐ P.v ☐ Mixed ☐ RDT (-)ve	
Treatment			
6.1.18.	Correct treatment<br>P.f - ACT × 3 days and PQ × 1st day<br>P.v - CQ × 3 days and PQ × 14 days<br>Mixed - ACT × 3 days and PQ × 14 days<br>RDT (-)ve – Refer to BHS	☐ Yes<br>☐ No	
Health education			
6.1.19.	Counselling and health education	☐ Yes<br>☐ No	Topics<br>☐ Signs and symptoms<br>☐ Mode of transmission<br>☐ Danger signs<br>☐ Treatment regime<br>☐ Importance of taking drug to full course<br>☐ DOTS<br>☐ Preventive measures<br>☐ Others …………………………

**6.2.**	**Dengue fever**		
History taking and physical examnination			
6.2.1.	Asking the patients background information<br>(Name, age, sex, address)	☐ Yes<br>☐ No	
6.2.2.	Assessing signs and symptoms of dengue fever	☐ Yes<br>☐ No	
6.2.3.	Assessing danger signs	☐ Yes<br>☐ No	
6.2.4.	Measuring the body temperature correctly	☐ Yes<br>☐ No	
Pre-referral treatment			
6.2.5.	Prescribing correct dose of Paracetamol according to age	☐ Yes<br>☐ No	
6.2.6.	Prescribing correct dose of ORS according to age	☐ Yes<br>☐ No	

Health education			
6.2.7.	Counselling and health education	☐ Yes<br>☐ No	Topics<br>☐ Signs and symptoms<br>☐ Mode of transmission<br>☐ Danger signs<br>☐ Prevention from mosquito bite<br>☐ Larva control<br>☐ Other …………………………

**6.3.**	**TB**		
History taking and physical examination			
6.3.1.	Asking the patients background information<br>(Name, age, sex, address,)	☐ Yes<br>☐ No	
6.3.2.	Assessing signs and symptoms of TB	☐ Yes<br>☐ No	
6.3.3.	Assessing TB suspected patients in the family	☐ Yes<br>☐ No	
6.3.4.	Measuring body temperature correctly	☐ Yes<br>☐ No	
Pre-referral treatment			
6.3.5.	Prescribing correct dose of Paracetamol according to age.	☐ Yes<br>☐ No	
Health education			
6.3.6.	Counselling and health education	☐ Yes<br>☐ No	Topics<br>☐ Signs and symptoms<br>☐ Mode of transmission<br>☐ Preventive measures<br>☐ Others …………………………

**6.4.**	**Childhood diarrhoea**		
6.4.1.	Asking the patients background information<br>(Name, age, sex, address)	☐ Yes<br>☐ No	
6.4.2.	Assessing signs and symptoms of diarrhoea (frequency, type)	☐ Yes<br>☐ No	

6.4.3.	Assessing signs of dehydrations	☐ Yes<br>☐ No	
Pre-referral treatment			
6.4.4.	Prescribing correct dose of zinc tablet according to age	☐ Yes<br>☐ No	
6.4.5.	Prescribing correct dose of ORS according to age	☐ Yes<br>☐ No	
Health education			
6.4.6.	Counselling and health education	☐ Yes<br>☐ No	Topics<br>☐ Signs and symptoms<br>☐ Mode of transmission<br>☐ Hygiene (hand, water, food, latrine)<br>☐ Benefits of zinc and ORs<br>☐ ORS preparation method<br>☐ Preventive measures<br>☐ Risk of using antibiotics<br>☐ Others …………………………

6.5.	Other febrile illnesses(RDT negative fever)		
History taking and physical examination			
6.5.1.	Asking the patients background information<br>(Name, age, sex, address)	☐ Yes<br>☐ No	
6.5.2.	History taking of fever	☐ Yes<br>☐ No	
6.5.3.	Measuring body temperature correctly	☐ Yes<br>☐ No	
Pre-referral treatment			
6.5.4.	Prescribing correct dose of Paracetamol according to age	☐ Yes<br>☐ No	

Health education			
6.5.6.	Counselling and health education	☐ Yes<br>☐ No	Topics<br>☐ Febrile illnesses<br>☐ Measures to lower body temperature<br>☐ Others …………………………….

6.6.	**Recording**		
6.6.1.	There is correct recording in relevant registers<br>☐ Carbonless malaria registers	☐ Correct<br>☐ Incorrect	
	☐ ICMV daily register	☐ Correct<br>☐ Incorrect	
	☐ CIME record book	☐ Correct<br>☐ Incorrect	
	☐ Referral form	☐ Correct<br>☐ Incorrect	
	☐ Malaria CI and classification form	☐ Correct<br>☐ Incorrect	
	☐ ICMV quarterly report	☐ Correct<br>☐ Incorrect	
	☐ အခြား	☐ Correct<br>☐ Incorrect	

**6.7.**	**Patient referral**		
6.7.1.	Patients are referred to health facility according to guidelines. (Danger signs, Pregnancy, Infants, TB, Dengue haemorrhagic fever, childhood diarrhoea and other febrile illnesses)	☐ Yes<br>☐ No	
6.7.2.	Fill the referral form completely in patient referral	☐ Yes<br>☐ No	
**6.8.**	**Malaria surveillance**		
6.8.1.	Malaria cases are notified within 24 hours after diagnosis	☐ Yes<br>☐ No	
6.8.2.	How the malaria cases are notified.	☐ Telephone call<br>☐ SMS<br>☐ Viber (or) Messenger<br>☐ Other …………………………………………….	
6.8.3.	Difficulties in notification of malaria cases	Please mention:	
6.8.4.	Volunteer carries out initial case investigation of malaria cases	☐ Yes<br>☐ No	
6.8.5.	Difficulties in malaria case investigation	☐ Section A: Basic information of patient<br>☐ Section B: Case classification<br>☐ Section C: Analysis of forward transmission<br>☐ Section D: Reactive case detection<br>☐ Section E: Case classification (Summary)	

6.8.6.	If there are any difficulties, please mention.		
6.8.7.	Volunteer assists in foci investigation and reactive case detection.	☐ Yes<br>☐ No	If "Yes", how?
**6.9**	**Assistant in disease control and TB DOTS activities**		
6.9.1.	Educating or distributing pamphlets about disease prevention measures	☐ Yes<br>☐ No	If "Yes", what is the topics?
6.9.2.	What kind IEC materials does the volunteer have?	☐ Poster<br>☐ Pamphlet<br>☐ Other ………………………	
6.9.3.	Other disease control activities	☐ Distributing insecticide treated net<br>☐ Indoor spraying of insecticide<br>☐ Larva control activities<br>☐ Others …………………………………………	
Larva control			
6.9.4.	Volunteer carries out larva control activities.	☐ Yes<br>☐ No	If "Yes", how many households?<br>………………………………………………………………
6.9.5.	Please mention the larva control activities carried out		
Sputum collection and TB DOTS			
6.9.6.	Volunteer assists in sputum collection.	☐ Yes<br>☐ No	If "Yes", how?<br>………………………………………………………………
6.9.7.	Volunteer carries out TB DOTS.	☐ Yes<br>☐ No	If "Yes", how many patients?

6.9.8	Volunteer prescribes anti-TB drugs correctly.	☐ Yes<br>☐ No	
**6.10.**	**Health education**		
Setting			
6.10.1.	Place for health education section		
6.10.2.	Method of health education	☐ Group discussion<br>☐ Lecture<br>☐ Informal talk	
6.10.3.	Aids and facilities used in health education	☐ Poster<br>☐ Pamphlet<br>☐ Flip chart<br>☐ Other	
Attendants			
6.10.4.	Number of attendants		
6.10.5.	Type of attendants (Gender, age, type of population e.g., MMP)		

Content		Topics
6.10.6.	☐ Malaria	☐ Sign and symptoms<br>☐ Mode of transmission<br>☐ Danger signs<br>☐ Treatment<br>☐ Importance of taking drug to full course

		☐ Preventive measures<br>☐ Others ……………………………….
6.10.7.	☐ Dengue haemorrhagic fever	☐ Signs and symptoms<br>☐ Mode of transmission<br>☐ Danger signs<br>☐ Pre-referral treatment<br>☐ Prevention from mosquito bite<br>☐ Larva control<br>☐ Other…………………………………….
6.10.8.	☐ TB	☐ Signs and symptoms<br>☐ Mode of transmission<br>☐ Danger signs<br>☐ Pre-referral treatment<br>☐ Preventive measures<br>☐ Other …………………………………….
6.10.9.	☐ Childhood diarrhoea	☐ Signs and symptoms<br>☐ Mode of transmission<br>☐ Danger signs<br>☐ Pre-referral treatment<br>☐ Benefits of zinc and ORS<br>☐ ORS preparation<br>☐ Preventive measures<br>☐ Risks of using antibiotics<br>☐ Others ………………………………
6.10.10.	Is the health education by volunteer understandable?	
6.10.11.	Can the volunteer lead the heath education session actively?	
End of session		
