# Supplementary material for: Acceptability, feasibility and fidelity of an expanded role for community health workers for malaria elimination in Myanmar: A mixed-method study
Source: PLOS Glob Public Health. 2025 Aug 13;5(8):e0004986. doi: 10.1371/journal.pgph.0004986 (PMC12349089; doi:10.1371/journal.pgph.0004986)
Supplement: S4 — (PDF) [file pgph.0004986.s019.pdf]

Supervision checklist

Supervision Checklist for Community Based Integrated Malaria Elimination (CIME) Volunteer Activities

This is the supervision checklist for CIME volunteer's supervisors. The supervision is expected to be conducted in about 2 hours.

**1. Background Information**					
1.1.	State/ Region:		1.2.	Township:	
1.3.	Name of volunteer:		1.4.	Village name:	
1.5.	Name of RHC:		1.6.	Name of sub-center	
1.7.	Starting time:		1.8.	Ending time:	
1.9.	Date of supervision (DD/MM/YYYY):				
**Setting**					
1.10.	CIME signboard in visible place	Yes ☐ No ☐<br>☐ Not seen			
1.11.	Where does CIME volunteer usually see the patient?	☐ Adequate ventilation<br>☐ Adequate lighting<br>☐ Adequate privacy<br>☐ Other			

**2. Records, Reports and References**				
		Items		Reasons for "Absent"
2.1.	Presence of records, reports and references	1. ☐ CIME volunteer manual<br>2. ☐ Malaria carbonless register<br>3. ☐ ICMV daily register<br>4. ☐ CIME record book<br>5. ☐ Referral form<br>6. ☐ Malaria case investigation and classification form<br>7. ☐ ICMV quarterly report<br>8. ☐ Other ……………………		
Record reviewing for previous 2 weeks				
Malaria Carbonless Register		Number	Remarks	
2.2.	Number of patients RDT tested			
2.3.	Number of P.f positive patient			
2.4.	Number of P.v positive patient			
2.5.	Number of Mixed patients			

2.6.	Number of malaria patient referral		
2.7.	Reasons for referral	☐ Danger signs ☐ Pregnant mother<br>☐ Infant ☐ Other	
ICMV Daily Register		Number	Remarks
2.8.	Number of patients attended		
2.9.	Number of RDT (-ve) fever		
2.10.	Number of Childhood Diarrhoea		
2.11.	Number of TB suspected patient		
2.12.	Number of Dengue suspected patient		
2.13.	Number of referrals made		If all suspected cases are not referred, please ask the reasons and describe the solutions made.<br>……………………………………………………………<br>……………………………………………………………<br>……………………………………………………………
CIME record book		Number	Remarks
2.14.	Number HE session		
2.15.	Number of participants attended in HE session		
2.16.	Number of malaria case notifications within 24 hours		If all malaria positive cases are not notified within 24 hours, please ask the reasons and solution made.<br>……………………………………………………<br>……………………………………………………<br>……………………………………………………
2.17.	Number of initial case investigations and classification by CIME		If all malaria positive cases are not investigated, please ask the reasons and describe the solution made.<br>…………………………………………………....<br>……………………………………………………<br>……………………………………………………
2.18.	Number of malaria patient provided DOT		
2.19.	All records are used correctly.	Yes ☐ No ☐	Which records are not used correctly? How the records are not used correctly?
2.20.	Submit reports regularly	Yes ☐ No ☐	

2.21.	Records, reports and references are kept in safe place (dedicated box or bag, place where the volunteer can only have access)	Yes ☐ No ☐	
2.22.	Any difficulties with recording and reporting	Yes ☐ No ☐	Please mention.
2.23.	How do you solve as a supervisor?		

**3. Logistic Management**					
Medicines and commodities		Remaining balance	Expired date	Is there stock out during last 2 weeks?	If yes, how many days?
3.1.	RDT			Yes ☐ No ☐	days
3.2.	RDT Ziplock bag			Yes ☐ No ☐	days
3.3.	ACT			Yes ☐ No ☐	days
3.4.	CQ			Yes ☐ No ☐	days
3.5.	PQ			Yes ☐ No ☐	days
3.6.	Paracetamol			Yes ☐ No ☐	days
3.7.	Multivitamin			Yes ☐ No ☐	days
3.8.	Zinc			Yes ☐ No ☐	days
3.9.	ORS			Yes ☐ No ☐	days
3.10.	Enough balance of RDT and antimalarial	Yes ☐ No ☐	If the balances are not enough for next 2 weeks, please refill.		
Recording		Tick	How it is recorded and reasons for “No”.		
3.11.	Presence of Stock book	Yes ☐ No ☐			
3.12.	Correctly recorded	Yes ☐ No ☐			
3.13.	Recorded up to date	Yes ☐ No ☐			
3.14.	Consistent with usage and patient	Yes ☐ No ☐			
3.15.	Consistent with ground balance	Yes ☐ No ☐			
Proper storage		Tick	If yes, corrections made by supervisor.		
3.16.	Store properly in safe place (Box, bag)	Yes ☐ No ☐			
3.17.	Enough space to store the stocks (Shelf, box)	Yes ☐ No ☐			
3.18.	Far from Heat, Sunlight, Rain/Humidity	Yes ☐ No ☐			
3.19.	RDT and medicines are damaged and/or changed in color.	Yes ☐ No ☐			
3.20.	Lancet and blood contaminated materials are disposed into the safety box properly	Yes ☐ No ☐			

Supported package			
Items		Tick	Current situation and reasons
3.21.	CIME Backpack/ Plastic box	Yes ☐ No ☐	
3.22.	Thermometer	Yes ☐ No ☐	
3.23.	Weighing machine	Yes ☐ No ☐	
3.24.	Torch light	Yes ☐ No ☐	
3.25.	Malaria treatment chart	Yes ☐ No ☐	
3.26.	Flipchart and poster, pamphlet	Yes ☐ No ☐	
3.27.	Pen to record on RDT	Yes ☐ No ☐	
3.28.	Ziplock bag for RDT storage	Yes ☐ No ☐	
3.29.	Code sticker for RDT	Yes ☐ No ☐	

**CIME volunteer's knowledge assessment and observation**				
**4. Malaria**				
4.1.	Knowledge assessment	Volunteer's response		
4.1.1.	Please mention signs and symptoms.			
4.1.2.	Please mention mode of transmission.			
4.1.3.	How can malaria be prevented?			
4.1.4.	Please mention the signs and symptoms of severe malaria.			
4.1.4.	What are the testing criteria for RDT testing			
4.2.	Observation of RDT testing	Correct	Incorrect	Not applicable
4.2.2.	RDT preparation			
4.2.3.	Patient preparation			
4.2.4.	Blood collection & dispensing			
4.2.5.	Timing and reading results			
4.2.6.	Recording results			
4.2.7.	Disposal of infectious material			
4.2.8.	Delivering result			
4.3.	Observation of Case Management	Yes	No	Not applicable
4.3.1.	Assess the signs and symptoms of malaria			
4.3.2.	Assess the all signs of severe malaria			
4.3.3.	RDT testing			
4.3.4.	Giving the correct treatment			
4.3.5.	Counsel (correct messages on full dose, full course)			
4.3.6.	Refer to health facility if there is signs and symptoms of severe malaria, infant and pregnant mother			
4.3.7	Notify malaria positive cases within 24 hours			

4.3.8	Conduct initial malaria case investigation and classification by CIME volunteer			
4.3.9.	Conduct DOT for malaria positive patient			
4.4.	Questions for Malaria treatment technical competencies	Correct	Incorrect	Not applicable
4.4.1	A 36 years old man comes to you because of fever. The RDT test result shows PF and PV mixed infection. What will be the treatment for him?			
4.4.2	A four years old boy presented to you with fever. The RDT test shows positive PV infection. What will be the treatment for him?			
4.4.3	A 12-year-old girl presented to you for fever and the RDT test results show positive PF infection. What will be the treatment for her?			
4.4.4	A pregnant mother comes to you for fever and the RDT results show positive PF infection. What will you do?			

**5. Dengue**				
5.1.	Knowledge assessment	Volunteer's response		
5.1.1.	Please mention signs and symptoms.			
5.1.2.	Please mention mode of transmission.			
5.1.3.	How can dengue be prevented?			
5.1.4.	Please mention the Danger signs of Dengue.			
5.2.	Observation of Case Management	Yes	No	Not applicable
5.2.1.	Assess the signs and symptoms of dengue			
5.2.2.	Assess the danger signs			
5.2.3.	Measure body temperature			
5.2.4.	Give paracetamol according to age			
5.2.5.	Give ORS according to age			
5.2.6.	Counsel (correct messages on danger signs, feeding, personal protective measures, larva control)			
5.2.7.	Refer to health facility			

**6. Tuberculosis**				
6.1.	Knowledge assessment	Volunteer's response		
6.1.1.	Please mention signs and symptoms.			
6.1.2.	Please mention mode of transmission.			
6.1.3.	How can TB be prevented?			
6.1.4.	What are the treatment options for TB?			
6.2.	Observation of Case Management	Yes	No	Not applicable

6.2.1.	Assess the signs and symptoms of TB			
6.2.2.	Assess history of suspected TB patient in family members			
6.2.3.	Measure body temperature			
6.2.4.	Give paracetamol according to age			
6.2.5.	Counsel (correct messages on signs and symptoms, transmission, treatment and preventive measures)			
6.2.6.	Refer to health facility			

**7. Childhood diarrhea**				
7.1.	Knowledge assessment	Volunteer's response		
7.1.1.	Please mention signs and symptoms.			
7.1.2.	Please mention mode of transmission.			
7.1.3.	How can diarrhoea be prevented?			
7.1.4.	How to provide ORS and Zinc according to age.			
7.2.	Observation of Case Management	Yes	No	Not applicable
7.2.1.	Assess the signs and symptoms of diarrhea.			
7.2.2.	Assess signs of dehydration			
7.2.3.	Give Zinc according to age			
7.2.4.	Give ORS according to age			
7.2.5.	Counsel (correct messages on signs and symptoms, transmission and preventive measures)			
7.2.6.	Refer to health facility			

**8. RDT (-) ve Fever**				
8.1.	Knowledge assessment	Volunteer's response		
8.1.1.	Please mention causes of fever			
8.1.2.	Please mention measures to relieve fever			
8.2.	Observation of Case Management	Yes	No	Not applicable
8.2.1.	Give Paracetamol according to age			
8.2.2.	Counsel (correct messages on causes of fever, measures to relieve fever)			
8.2.3.	Refer to health facility			

8. Findings and solution made by supervisors	
**Findings**	**Solution made**
……………………………………………<br>……………………………………………<br>……………………………………………<br>……………………………………………<br>……………………………………………<br>……………………………………………	……………………………………………<br>……………………………………………<br>……………………………………………<br>……………………………………………<br>……………………………………………<br>……………………………………………

Remarks: Please bring RDT, antimalarials, ORS, Zinc, Paracetamol, Pamphlet and Poster when you make supervision visit to volunteer.

Volunteer signature: ………………………

Volunteer name: ………………………….

Village: ………………………………

Date: ………………………………………

Supervisor signature: ………………………

Supervisor name: ………………………….

Supervisor position: ………………………

Department: ………………………………

Date: ………………………………………
